# Patient‐Reported Outcomes of Palatopharyngeal Surgery Without Tonsillectomy: A Meta‐Analysis

**DOI:** 10.1002/lary.70526

**Published:** 2026-03-29

**Authors:** Samuel Tschopp, Flora Meinert, Georgios Mantokoudis, Marco Caversaccio, Urs Borner

**Affiliations:** ^1^ Department of Otorhinolaryngology Head and Neck Surgery, Inselspital, University Hospital and University of Bern Bern Switzerland

**Keywords:** obstructive sleep apnea, palatal surgery, patient‐reported outcome measure, PROM, sleep surgery, uvulopalatopharyngoplasty

## Abstract

**Objective:**

To compare patient‐reported outcome measures (PROMs) across different palatopharyngeal surgical modifications without tonsillectomy for snoring and obstructive sleep apnea (OSA).

**Data Sources:**

MEDLINE, Embase, Web of Science, ClinicalTrials, CINAHL, Cochrane Library, ICTRP.

**Review Methods:**

We conducted a systematic review and meta‐analysis, including original studies of adult patients undergoing palatopharyngeal surgery without tonsillectomy for snoring and OSA. To reduce confounding, we excluded tonsillectomy, as tonsil size is a major predictor of outcome. Primary PROMs included changes in daytime sleepiness, as measured by the Epworth Sleepiness Scale (ESS), and snoring intensity, assessed on a visual analog scale, analyzed by surgical technique using random‐effects models.

**Results:**

Fifty‐five studies with 1815 patients were included. Overall, palatopharyngeal surgery without tonsillectomy reduced ESS by a mean of 3.3 points (95% CI, 2.7 to 3.9) and snoring intensity by 4.1 points (95% CI, 3.7 to 4.6). Among surgical techniques, suture palatopharyngoplasty, muscle relocation, cold‐steel techniques, and powered instruments showed the largest reductions in both daytime sleepiness and snoring. Meta‐regression analyses showed no evidence for declining treatment effectiveness with longer follow‐up durations for both PROMs. Heterogeneity across studies was considerable, with a moderate overall risk of bias.

**Conclusion:**

Palatopharyngeal surgery without tonsillectomy effectively improves patient‐reported daytime sleepiness and snoring intensity, with outcomes varying by surgical techniques. Suture palatopharyngoplasty, muscle relocation, cold‐steel techniques, and the use of powered instruments appear to be most effective in improving PROMs. These results inform surgical planning and counseling in patients seeking surgical treatment for snoring and daytime sleepiness.

**Trial Registration:** PROSPERO: CRD42024559063.

## Graphical abstract

Palatopharyngeal surgery without tonsillectomy leads to clinically meaningful improvements in daytime sleepiness and snoring, but patient‐reported outcomes vary markedly by surgical technique. Suture‐based procedures, muscle relocation, cold‐steel approaches, and powered instruments show the largest and durable benefits, with no evidence of effect waning over time. These findings underscore that how the palate is modified matters and should be considered in the surgical management of snoring and obstructive sleep apnea.

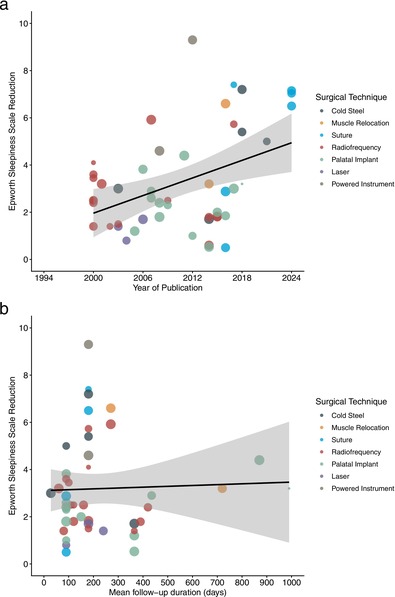

## 1 Introduction

Patient‐reported outcome measures (PROMs) are essential for evaluating surgical treatments from a patient‐centered perspective [1, 2, 3]. Although multiple PROMs exist for snoring and obstructive sleep apnea (OSA) surgery, many are insufficiently validated [1], and no consensus on their use has been reached [1, 2, 3]. This lack of standardization limits comparability across studies using PROMs for patient‐centered care [1], so outcome studies often emphasize objective metrics such as the apnea‐hypopnea index (AHI). While AHI reductions following palatopharyngeal surgery are well documented, many patients, particularly those with primary snoring or mild disease, prioritize symptomatic improvements over objective physiological measures [4, 5, 6]. A decrease in snoring intensity and relief of daytime sleepiness are often primary reasons for undergoing surgery. Excessive daytime sleepiness can compromise daily activities and overall quality of life [7, 8, 9]. Snoring can have a profound impact on both the person experiencing it and those around them [10, 11].

Palatopharyngeal surgery is frequently performed to treat snoring and OSA with generally proven effectiveness [4, 5, 12]. Fujita et al. first described the uvulopalatopharyngoplasty (UPPP) in 1981 [13]. The classical UPPP involved tonsillectomy and removal of soft tissue in the oropharynx, followed by approximation with resorbable sutures. Over time, several modifications to this technique have been described to stabilize the oropharyngeal airway during sleep. They focus on muscle relocation rather than tissue resection [12, 14] and often employ technological advances, such as radiofrequency ablation, laser, powered instruments, and implants. These modifications aim to improve effectiveness and palatopharyngeal function while reducing tissue removal [12, 14] and complications such as foreign body sensation, velopharyngeal insufficiency, and dry mouth [15]. Additionally, modern modifications are increasingly performed as outpatient procedures, reducing hospital stays, sick days, and healthcare costs.

The overall effectiveness of palatopharyngeal surgery in improving both objective and subjective outcomes has been well demonstrated [4, 5, 12]. We have previously shown the technique‐dependent differences in the effectiveness of palatopharyngeal surgery in reducing AHI [6]. To date, no comparative analysis has specifically evaluated palatopharyngeal surgical modifications regarding PROMs. Given the central role of PROMs in patient‐centered care and surgical decision‐making for patients with snoring and OSA, understanding the comparative effectiveness of these techniques on symptom outcomes is essential for informed patient counseling and surgical planning. Accordingly, this meta‐analysis compares palatopharyngeal surgery modifications without tonsillectomy with respect to daytime sleepiness and snoring intensity, providing evidence to guide treatment decisions.

## 2 Methods

Reporting for this systematic review and meta‐analysis adheres to the Preferred Reporting Items for Systematic Reviews and Meta‐Analyses (PRISMA) guidelines, and the study was prospectively registered in the PROSPERO database (CRD42024559063) [16]. We included studies of adult patients with snoring or OSA (population) undergoing palatopharyngeal surgery without tonsillectomy (intervention). Surgical techniques were grouped according to procedural characteristics (comparison), and changes in PROM for daytime sleepiness and snoring intensity (outcome) were analyzed. For methodological consistency and comparability across studies, we limited inclusion to the most commonly used and widely accepted PROMs in sleep surgery, the Epworth Sleepiness Scale (ESS) [17] for daytime sleepiness and a 10‐point visual analog scale for snoring.

### 2.1 Data Sources and Search Strategy

A systematic search was conducted across MEDLINE, Embase, Web of Science Core Collection, ClinicalTrials, CINAHL, Cochrane Library, and the WHO International Clinical Trials Registry Platform (ICTRP), and was last updated in December 2025. The search strategy was developed in collaboration with a medical information specialist from the University of Bern. The search strategy targeted two main concepts: (1) the intervention—palatopharyngeal surgical procedures and their modifications; and (2) the population—adult patients with snoring or obstructive sleep apnea, explicitly excluding pediatric and animal studies. The detailed search string is given in the Supporting Information.

### 2.2 Study Selection

Two independent reviewers (S.T. and F.M.) screened the abstracts (Table S1) and full texts (Table S2) using predefined inclusion and exclusion criteria in the Covidence systematic review software (Veritas Health Innovation, Melbourne, Australia). Discrepancies were resolved by a third reviewer (U.B.).

For the abstract screening (Table S1), we included all articles presenting original data on palatopharyngeal outcomes in adult patients. Excluded were studies of multilevel or combined procedures and preselected patient populations, such as patients undergoing revision surgery. No exclusions were made based on the severity of sleep‐disordered breathing.

For the full‐text screening (Table S2), the same predefined eligibility criteria were applied. We included studies that reported either daytime sleepiness on the Epworth Sleepiness Scale [17] or snoring intensity on a 10‐point visual analog scale. We did not include studies that reported snoring intensity on scales other than a 10‐point scale or daytime sleepiness other than using the Epworth Sleepiness Scale [17]. These two PROMs were chosen because they are the most frequently used, ensuring sufficient data, improving comparability across studies, and reducing heterogeneity. Studies that failed to describe the surgical technique, lacked systematic patient recruitment, or had no accessible full text were also excluded. To control for tonsil size as a confounding factor, studies involving concomitant tonsillectomy were excluded [18, 19]. Likewise, studies that failed to distinguish between surgical techniques or that reported only on combined procedures were excluded. In studies with multiple treatment arms, all eligible treatment groups meeting the inclusion criteria were analyzed.

### 2.3 Data Extraction

Data were collected into a structured table, which all authors approved. The extraction was performed by two reviewers (S.T. and F.M.). For studies with multiple treatment arms, data from each group meeting the inclusion criteria were collected separately. Surgical techniques were analyzed and categorized into the following groups: cold‐steel, muscle relocation techniques, suture techniques, radiofrequency ablation, palatal implants, laser, and powered instruments. Cold‐steel procedures were defined as techniques that use traditional instruments without powered assistance, except for bipolar coagulation for hemostasis. Muscle relocation techniques comprise surgeries that reposition, advance, or remodel the palate or pharyngeal muscles, such as expansion sphincteroplasty. Suture techniques employ sutures to tension and stabilize soft tissues, including barbed‐suture palatopharyngoplasty. Radiofrequency procedures apply radiofrequency energy to induce submucosal fibrosis and stiffening. Laser procedures involve the ablation or reshaping of palatal tissue using laser energy. The powered instrument group encompassed surgeries that employed powered devices, such as microdebriders, ablative devices, and coblation‐based instruments. All palatal implants were analyzed as a single group. In practice, individual surgeons may introduce minor variations within a given technique, adapting their approach to each patient. Furthermore, techniques may overlap; for example, limited use of powered instruments may accompany a primarily cold‐steel procedure.

The primary outcomes were the absolute changes in daytime sleepiness, as measured by the Epworth Sleepiness Scale (ESS) [17], and snoring intensity, measured on a 10‐point visual analog scale. For studies reporting outcomes at multiple time points, PROMs from the longest follow‐up duration were chosen. Mean and standard deviation (SD) values were extracted directly. If effect sizes were presented using alternative metrics, mean differences and SDs were estimated from the available data when feasible [20].

### 2.4 Risk of Bias Assessment

The risk of bias was assessed using the risk of bias in nonrandomized studies of interventions (ROBINS‐I) tool across seven domains [21]. Using structured signaling questions, all studies were evaluated, and the seven domains were graded, as presented in Table S3.

### 2.5 Statistical Analysis

All analyses and visualizations were conducted using R (version 4.5.2, R Foundation for Statistical Computing, Vienna, Austria). The “meta” package was applied where appropriate to perform meta‐analyses [22]. Outcomes were reported as mean differences with 95% confidence intervals (CIs). Heterogeneity was assessed using the *I* ^2^ statistic. To explore heterogeneity, a subgroup analysis was conducted stratified by surgical technique. Due to anticipated heterogeneity across studies, pooled estimates were calculated using random‐effects models with a restricted maximum‐likelihood estimator. Meta‐regression was conducted to assess the association between PROMs and both follow‐up duration and publication date. Publication bias was evaluated using contour‐enhanced funnel plots.

## 3 Results

### 3.1 Search Yield and Study Selection

**FIGURE 1:**
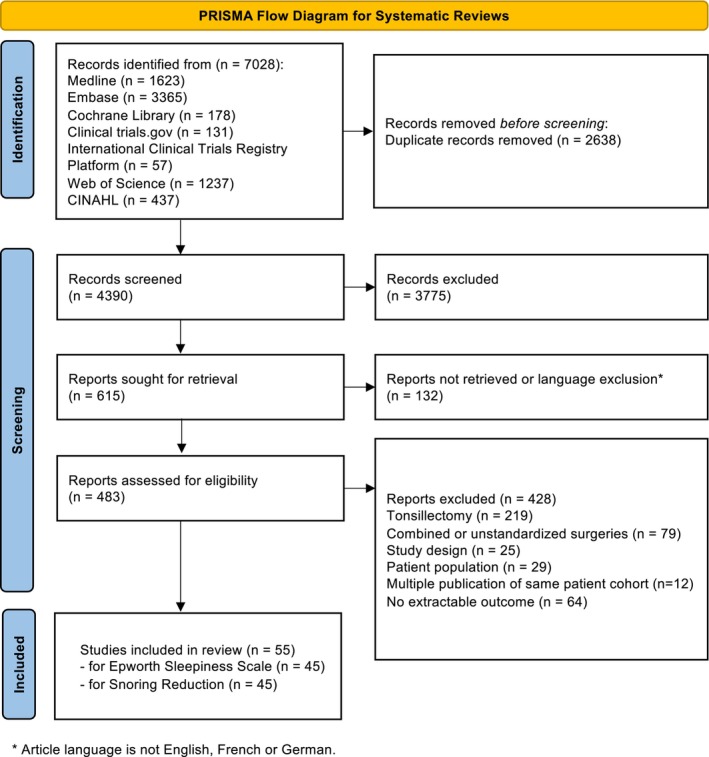
PRISMA flow diagram for systematic reviews. [Color figure can be viewed in the online issue, which is available at www.laryngoscope.com]

The automatic search yielded 3793 records for screening. After selection, 1815 patients from 55 articles, comprising 61 analyzable treatment groups, were included in this meta‐analysis (Figure 1). The inter‐rater agreement was moderate for the abstract (Cohen's kappa 0.55) and full‐text screening (Cohen's kappa 0.64). In two studies, two separate treatment groups could be included [23, 24]. One study evaluated snoring and ESS at different time points [25], and another article reported two subgroups of the same surgical technique, which could not be pooled for analysis [26]. The largest surgical groups were palatal implant (*n* = 562) and radiofrequency (*n* = 506), followed by muscle relocation (*n* = 219), cold‐steel (*n* = 181), suture palatopharyngoplasties (*n* = 143), laser (*n* = 133), and powered instruments (*n* = 71). Only four studies reported on in‐hospital procedures [24, 27, 28, 29], while all others described results from outpatient surgeries. Most studies were conducted in the United States (*n* = 11), followed by Germany (*n* = 6), Turkey (*n* = 6), and Taiwan (*n* = 5). Overall, the mean age was 42.8 years, with 69% of patients being male. The mean follow‐up duration was 416 days. The AHI reduced from preoperative 13.0/h to postoperative 7.7/h.

### 3.2 Daytime Sleepiness

**FIGURE 2:**
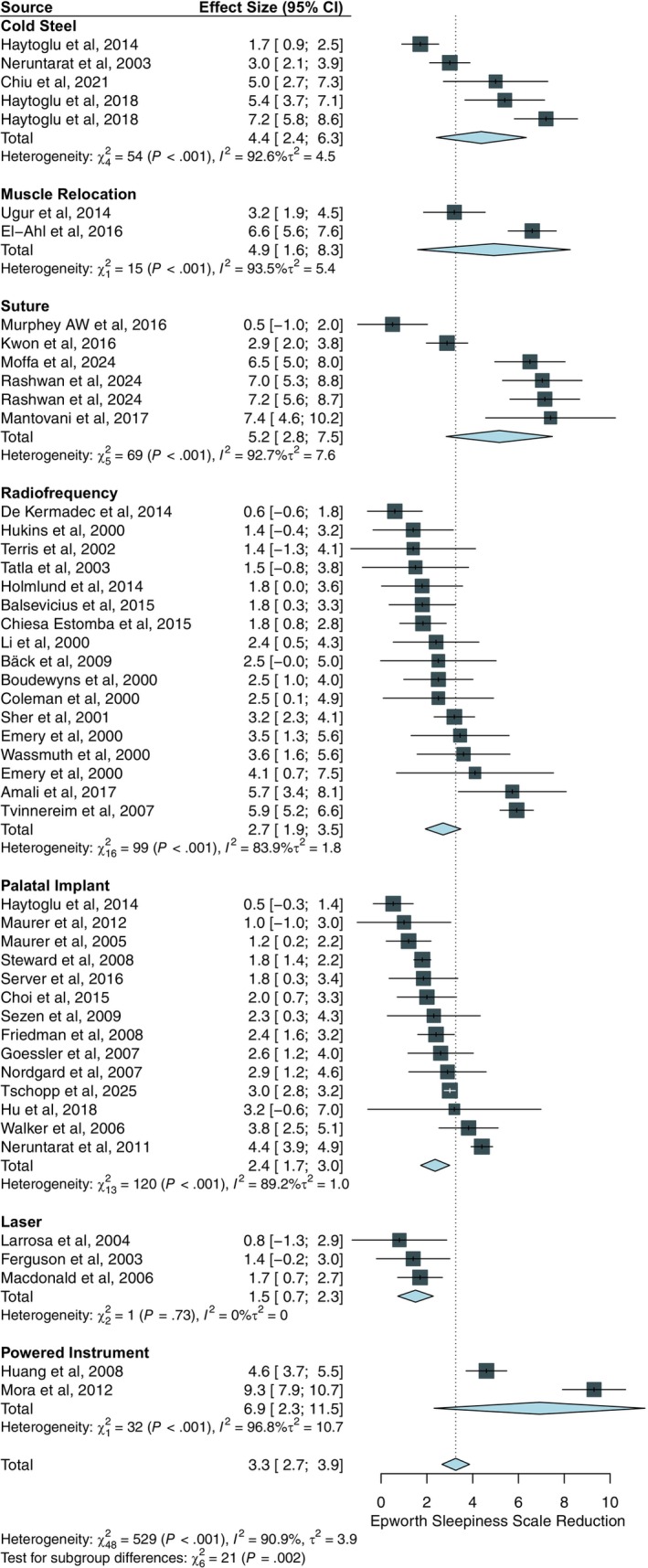
Forest plot for Epworth Sleepiness Scale reduction grouped by surgical technique. The reduction was defined as the absolute difference between pre‐ and postoperative measurements. [Color figure can be viewed in the online issue, which is available at www.laryngoscope.com]

**FIGURE 3:**
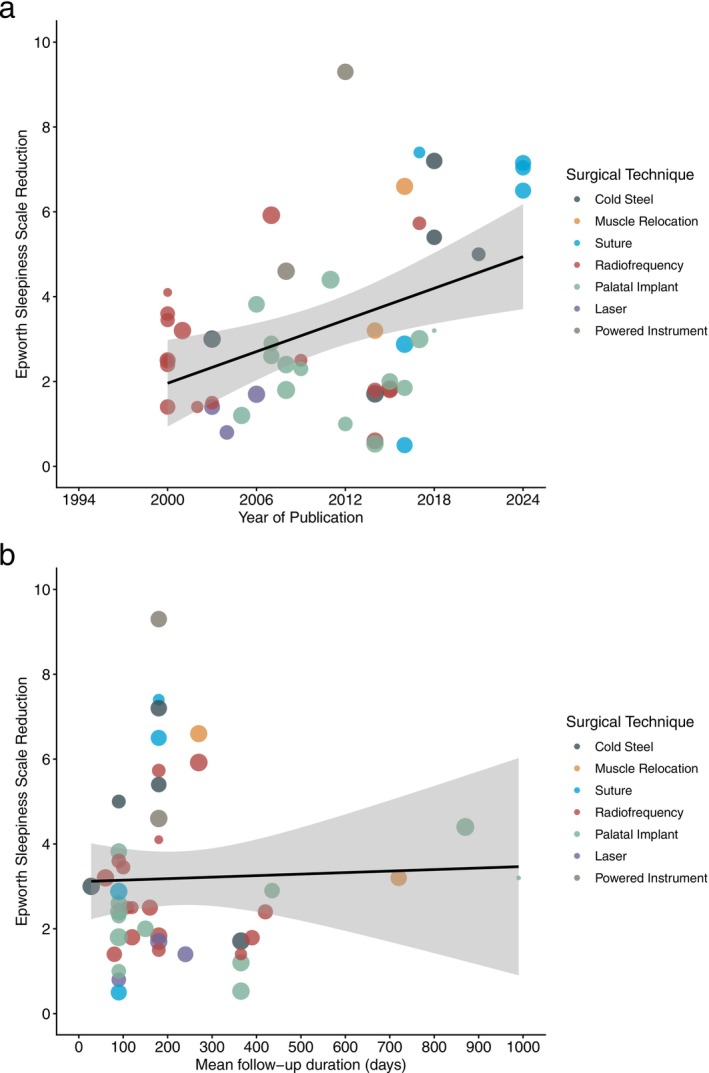
Epworth Sleepiness Scale reduction of palatal surgeries without tonsillectomy depending on the follow‐up duration (a) and publication date (b). The circle size indicates the weight of each study in the random effects meta‐analysis. A linear regression is represented as a solid line. [Color figure can be viewed in the online issue, which is available at www.laryngoscope.com]

Forty studies with 1505 patients reported daytime sleepiness outcomes (Table S4). Palatopharyngeal surgery without tonsillectomy reduced ESS by a mean of 3.3 points (95% CI, 2.7 to 3.9, Figure 2). The most considerable ESS reductions were observed with powered instruments (mean reduction 6.9, 95% CI, 2.3 to 11.5), suture palatopharyngoplasty (mean effect 5.2, 95% CI, 2.8 to 7.5), muscle relocation (mean effect 4.9, 95% CI, 1.6 to 8.3), and cold‐steel techniques (mean effect 4.4, 95% CI, 2.4 to 6.3). Only a minor effect was calculated for laser techniques (mean effect 1.5, 95% CI, 0.7 to 2.3). A meta‐regression revealed a slight improvement in the ESS reduction by publication date (estimate, 0.11; 95% CI, 0.04 to 0.19, *p* = 0.22, Figure 3a), indicating that newer publications do not demonstrate significantly better results. The treatment effect on the ESS reduction did not change with follow‐up duration (estimate, −0.001; 95% CI, −0.002 to 0.001, *p* = 0.22; Figure 3b). Placebo arms in seven studies demonstrated a small but significant reduction in ESS (mean effect 1.2, 95% CI, 0.5–1.8, Table S5 and Figure S1).

### 3.3 Snoring

**FIGURE 4:**
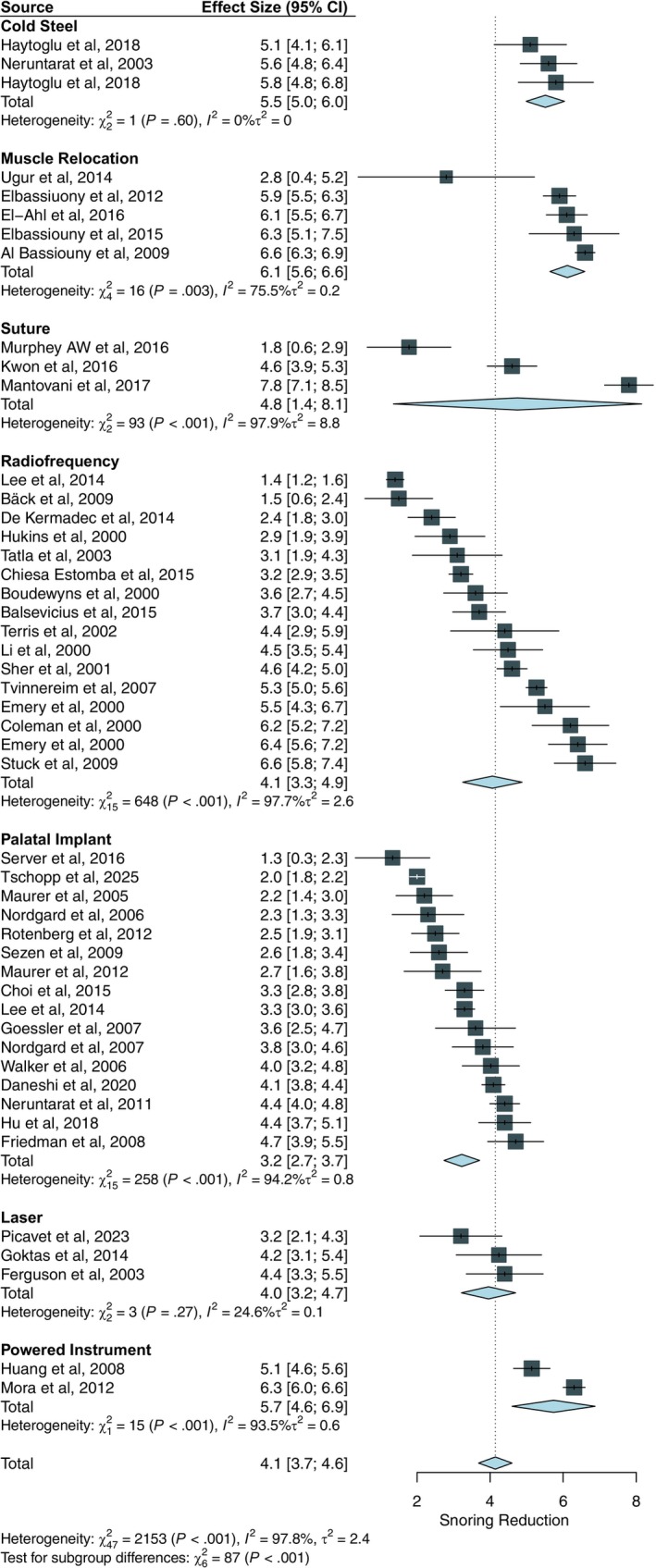
Forest plot for snoring reduction on a visual analog scale grouped by surgical technique. The reduction was defined as the absolute difference between pre‐ and postoperative measurements. [Color figure can be viewed in the online issue, which is available at www.laryngoscope.com]

**FIGURE 5:**
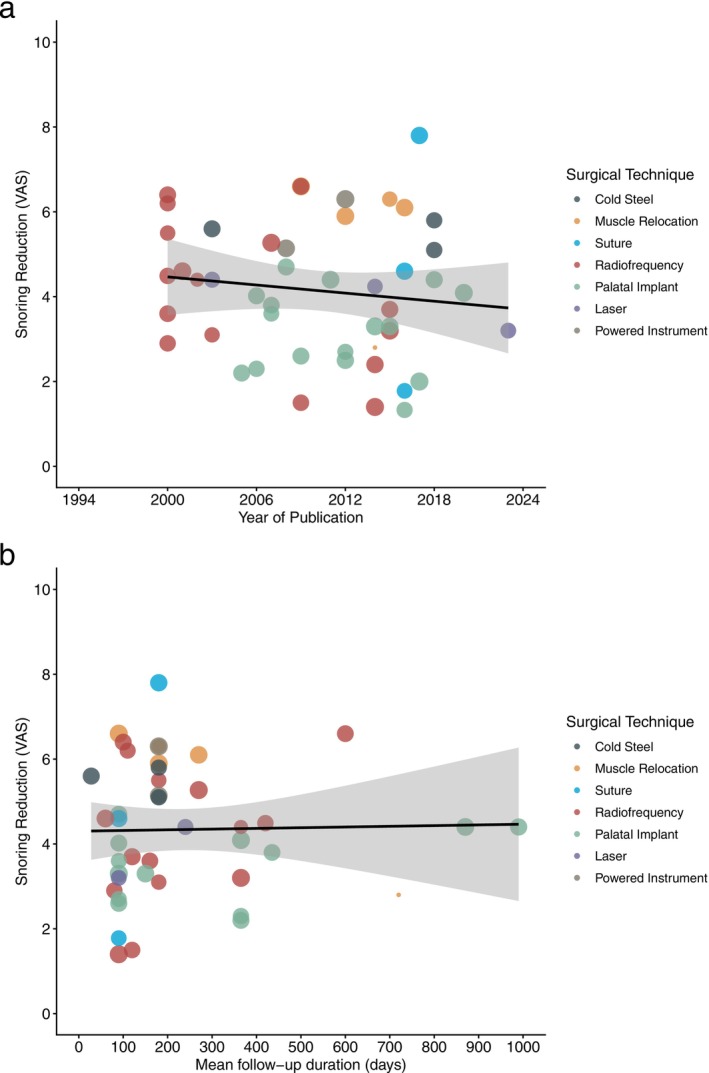
Snoring reduction on a visual analog scale (VAS) of palatopharyngeal surgeries without tonsillectomy depending on the follow‐up duration (a) and publication date (b). The circle size indicates the weight of each study in the random effects meta‐analysis. A linear regression is represented as a solid line. [Color figure can be viewed in the online issue, which is available at www.laryngoscope.com]

Forty‐two studies, including 1425 patients, were included in the analysis of snoring outcomes. Snoring intensity measured on a 10‐point visual analog scale decreased by a mean of 4.1 points (95% CI, 3.6 to 4.6, Figure 4). Similar to the reduction in daytime sleepiness, distinct differences were observed between surgical techniques. Muscle relocation (mean effect 6.1, 95% CI, 5.6 to 6.6), powered instruments (mean effect 5.7, 95% CI, 4.6 to 6.9), cold‐steel palatopharyngoplasty (mean effect 5.5, 95% CI, 5.0 to 6.0), and suture palatopharyngoplasty (mean effect 4.8, 95% CI, 1.4 to 8.1) demonstrated the most considerable reductions. Table S6 gives a summary of all studies included in the meta‐analysis for snoring. No evidence was found for an influence of follow‐up duration on the estimate (−0.001; 95% CI, −0.001 to 0.0001, *p* = 0.22, Figure 5a) or the publication date (estimate, −0.04; 95% CI, −0.11 to 0.03, Figure 5b) on snoring outcomes. Five placebo treatment arms demonstrated a modest reduction in snoring (mean effect 0.6, 95% CI, 0.3–0.8, Figure S2). Table S7 provides an overview of the placebo studies included in the analysis of snoring outcomes.

### 3.4 Risk of Bias Assessment

Using the RONINS‐I tool, most included studies were judged to have a moderate risk of bias across most domains (Figure S3). The classification of interventions was rated as having the lowest risk of bias, reflecting the clear inclusion criteria of the systematic review. In contrast, the most common sources of bias identified were confounding, primarily related to patient selection and disease severity, along with missing data and selection of reported outcomes. In some domains, these issues resulted in a serious risk of bias rating (Figure S4).

### 3.5 Publication Bias Assessment

Contour‐enhanced funnel plots were used to evaluate publication bias. Daytime sleepiness reduction displayed a slight asymmetry toward smaller effect sizes (Figure S5). The funnel plot for snoring reduction showed a symmetrical distribution with no apparent publication bias (Figure S6). Both graphs show substantial between‐study heterogeneity.

## 4 Discussion

This meta‐analysis demonstrates that palatopharyngeal surgery performed without tonsillectomy effectively reduces daytime sleepiness and snoring, although outcomes vary significantly across different surgical techniques. Techniques such as suture palatopharyngoplasty, muscle relocation, and the use of powered instruments were associated with greater reductions in ESS and snoring intensity, highlighting their potential advantages over other techniques in PROMs. These findings parallel those of our previous meta‐analysis evaluating technique‐dependent differences in AHI reduction [6]. Importantly, given that the included studies were predominantly observational and subject to moderate risk of bias, the results should not be interpreted as definitive evidence of the superiority of any single technique. In addition to PROMs and AHI outcomes, decisions regarding surgical technique should also incorporate adverse effects, complications, and individual patient characteristics and preferences.

We found no evidence that treatment effectiveness declined over follow‐up durations up to 3 years, challenging the commonly held assumption that the benefits of palatopharyngeal surgery diminish over time [30]. Furthermore, more recent publications have not shown systematically improved outcomes. This may reflect a trend toward less invasive surgical approaches with a more modest impact on PROM. However, this interpretation remains speculative, as we were unable to analyze temporal changes in individual surgical methods.

We deliberately excluded studies involving concomitant tonsillectomy because tonsil size is an established determinant of surgical outcome, and their inclusion would have introduced substantial clinical heterogeneity and confounding. This approach allowed a more focused comparison of technique‐specific effects among palatopharyngeal procedures by isolating the impact of palatopharyngeal modification itself. Several studies, including the randomized trial by Sundman et al., have underscored the critical role of tonsillectomy in determining surgical outcomes [18, 19, 31]. In our prior analysis of patients undergoing radiofrequency uvulopalatopharyngoplasty combined with tonsillectomy, tonsil size emerged as the strongest predictor of improvement in ESS scores and reduction in snoring [19]. These findings support the notion that tonsillectomy and the extent of tissue resection may be critical contributors to surgical success. Accordingly, the pronounced improvements observed with powered instruments may reflect a greater extent of tissue resection compared to other surgical approaches. In contrast, suture techniques and muscle relocation achieve similar improvements in PROMs, potentially with lower morbidity.

The literature PROM for OSA and snoring remains limited. Although several PROMs have been described, few have undergone robust validation [1, 2]. A recent review by Bhat et al. highlighted substantial deficiencies in the validation and reliability of frequently used PROMs [1]. Additionally, standard instruments such as the ESS and a visual analog scale for snoring intensity address only a narrow aspect of the disease burden, and broader disease‐specific quality‐of‐life dimensions are often not considered. Further, there is no consensus on their use in patients with OSA and snoring [1, 2, 3]. Few studies have sought to identify patient‐specific predictors of PROMs in palatopharyngeal surgery. Besides tonsil size, Erfanian et al. found in a small but well‐characterized cohort of patients that lower preoperative AHI and smaller neck circumference were associated with better postoperative patient satisfaction [19, 32]. Conversely, circumferential velar collapse in drug‐induced sleep endoscopy has been linked to poorer patient satisfaction [32]. Other studies found greater reductions in snoring among patients with lower body mass index [33].

Interpretation of PROM changes also requires consideration of the minimal clinically important difference (MCID), which determines if observed improvements are clinically meaningful to patients. For the ESS, Patel et al. [34] estimated an MCID of approximately two to three points, while Crook et al. [35] proposed an MCID of two points. Applying a threshold of two points, all evaluated techniques except laser palatopharyngoplasty achieved the MCID. For snoring, no MCID could be identified in the available literature, further emphasizing the need for consensus‐driven, validated PROMs. For visual analog pain scales, the MCID is estimated to be a reduction of 1 to 1.4 points [36]. In our meta‐analysis, all subgroups achieved this level of improvement.

Complications, adverse events, and time to return to a regular diet and normal activity are crucial factors to consider for any surgical technique. However, these data were not systematically reported and could not be formally compared in this meta‐analysis. Although definitions varied across studies, no serious adverse events were reported in the included articles. Radiofrequency palatopharyngoplasty studies predominantly report minor adverse events, including mucosal dryness, transient swallowing discomfort, and foreign‐body sensation [37, 38]. Complications such as palatal fistulas, hemorrhage, and loss of uvula were rare with radiofrequency techniques [37]. Compared with radiofrequency, laser techniques may be associated with more frequent swallowing disturbances, but otherwise demonstrate a similar adverse event profile [39]. Muscle relocation and suture techniques more commonly report wound dehiscence, velopharyngeal insufficiency, swallowing disturbances, and hemorrhage [40, 41, 42]. In addition, suture techniques were associated with granuloma formation and knot extrusion [41]. In one study, powered instrument techniques resulted in short‐term velopharyngeal insufficiency in 20% of patients and long‐term abnormal pharyngeal sensation in 12% [43]. Extrusion was a complication specific to palatopharyngeal implants, reported in 4%–11%, while other adverse events, such as bleeding, pain, and swallowing disturbances, appeared less frequent with this technique [44, 45, 46].

Thorough patient counseling remains the cornerstone to align surgical approaches with individual patient characteristics and preferences. We further advise caution in interpreting the apparent superiority of specific surgical subgroups. The effectiveness of AHI and PROM reduction does not account for other factors, such as complications, adverse effects, and patient‐specific anatomy and preferences. We emphasize that while significant differences among techniques were identified, these should not be overstated.

### 4.1 Limitations

Several limitations must be acknowledged. First, as surgical procedures were investigated, none of the included studies were blinded, and most were nonrandomized observational designs. Sample sizes were often small, and several studies excluded patients, such as those with severe obesity or severe OSA, thereby limiting generalizability despite these patients frequently representing typical candidates for palatopharyngeal surgery in routine clinical practice.

Second, patient distribution across surgical techniques was uneven, with a relative overrepresentation of palatal implants and radiofrequency procedures. Additionally, palatal implants are no longer available, rendering findings of this subgroup primarily of historical interest.

Third, to minimize confounding, studies involving tonsillectomy were excluded due to the well‐established effect of tonsil size on surgical outcomes [18, 19, 29]. Although this improved methodological homogeneity among the studies, it precluded the inclusion of techniques that inherently require tonsil removal, such as classical expansion sphincteroplasty. Additionally, it severely reduced the number of studies available for analysis. This exclusion may also have led to an underestimation of the overall treatment effect of palatopharyngeal surgery, as tonsillectomy is frequently performed in real‐world settings. Nevertheless, excluding tonsillectomy was considered justified to limit confounding and to better isolate technique‐specific effects on PROMs.

Fourth, the substantial heterogeneity was observed, likely reflecting variability in patient selection, surgical execution, and outcome assessment across studies. Since most studies report improvements, this heterogeneity is primarily driven by variability in effect size rather than the directionality of the effect. Although subgroup analyses partially reduced heterogeneity, clinically relevant variability persisted within individual technique categories, indicating that procedural categories alone do not capture important differences in patient characteristics and outcomes. This residual heterogeneity limits the interpretability of pooled estimates and underscores the need for standardized patient selection and outcome reporting, as well as more rigorous study designs. Furthermore, a direct comparison of techniques, or a network meta‐analysis, was not feasible due to the limited number of available publications.

Fifth, the frequency of specific surgical techniques may change over time. Modifications, such as barbed‐suture palatopharyngoplasty, are gaining popularity, whereas palatopharyngeal implants are no longer commercially available and are primarily of historical interest.

Finally, the meta‐analysis focused exclusively on daytime sleepiness assessed by the ESS and snoring on a 10‐point visual analog scale, as these PROMs are the most frequently reported and well‐standardized across studies. Although other disease‐specific PROMs are available, they were omitted because the data were too sparse to allow meaningful synthesis. In addition, broader disease‐related quality‐of‐life instruments that capture mental, physical, and social dimensions were not incorporated. Consequently, the relatively narrow set of disease‐specific PROMs analyzed may not fully capture the overall patient‐perceived benefit of palatopharyngeal surgery.

### 4.2 Strengths

To our knowledge, this is the first meta‐analysis specifically comparing PROMs across different palatopharyngeal surgical techniques with tonsillectomy. By excluding procedures involving tonsillectomy, we analyzed a more homogeneous patient cohort, improving the comparability of outcomes across studies. Despite this exclusion, we were able to include more than 1300 patients, providing a robust and clinically meaningful dataset for analysis. This meta‐analysis advances the understanding of the comparative effectiveness of palatopharyngeal surgery regarding PROMs, which are critical for treatment decisions in snoring and OSA. It provides surgeons and patients with practical, evidence‐based information for selecting surgical techniques that align with patient priorities, thereby supporting informed, patient‐centered care in clinical practice.

### 4.3 Future Implications

Future research should prioritize direct comparisons of contemporary palatopharyngeal surgical techniques within prospective, adequately powered, multiarm randomized controlled trials to robustly determine their relative effectiveness. Such trials should deliberately include procedures that require tonsillectomy to reduce selection bias and better reflect real‐world surgical decision‐making. In addition, PROMs, including quality‐of‐life measures, for OSA and snoring should be standardized to evaluate outcomes holistically. Future studies should systematically report PROMs, complications, and adverse effects, as these factors are critical for aligning treatment options with patient preferences.

## 5 Conclusion

Palatopharyngeal surgery without tonsillectomy effectively reduces daytime sleepiness and snoring intensity, with outcomes varying substantially across surgical techniques. Powered instruments, suture‐based procedures, cold‐steel palatopharyngoplasties, and muscle relocation techniques appear to provide the most pronounced improvements in PROMs. Further comparative research, preferably adequately powered multiarm randomized controlled trials, focusing on objective sleep metrics, quality‐of‐life measures, and complication rates, is essential to guide evidence‐based, patient‐centered care in the surgical management of snoring and obstructive sleep apnea.

## Funding

The authors have nothing to report.

## Ethics Statement

The authors have nothing to report.

## Conflicts of Interest

The authors declare no conflicts of interest.

## Supporting information


**Figure S1:** Forest plot for epworth sleepiness scale reduction of the placebo group. The reduction was defined as the absolute difference between pre‐ and postoperative measurements.
**Figure S2:** Forest plot for snoring reduction of the placebo group. The reduction was defined as the absolute difference between pre‐ and postoperative measurements.
**Figure S3:** Risk of bias summary plot.
**Figure S4:** Risk of bias traffic light plot.
**Figure S5:** Contour‐enhanced funnel plot for the epworth sleepiness scale reduction of the included studies.
**Figure S6:** Contour‐enhanced funnel plot for the snoring reduction of the included studies.
**Table S1:** Abstract review coding schema with the predefined inclusion and exclusion criteria.
**Table S2:** Full‐text article review coding schema with the predefined inclusion and exclusion criteria.
**Table S3:** Risk of bias assessment using the ROBINS‐I tool.
**Table S4:** Summary of studies included in the epworth sleepiness scale reduction meta‐analysis.
**Table S5:** Summary of placebo studies for the epworth sleepiness scale reduction.
**Table S6:** Summary of studies included in the snoring reduction meta‐analysis.
**Table S7:** Summary of placebo studies for the snoring reduction.
**Supporting Informations**. Systematic search syntax for all databases.

## Data Availability

The data that supports the findings of this study are available in the Supporting Information of this article.
