# Supplementary material for: Patient‐Reported Outcomes of Palatopharyngeal Surgery Without Tonsillectomy: A Meta‐Analysis
Source: Laryngoscope. 2026 Mar 29;136(9):3706–17. doi: 10.1002/lary.70526 (PMC13460715; doi:10.1002/lary.70526)
Supplement: Supplementary file 1 [file LARY-136-3706-s001.docx]

**SUPPLEMENTAL INFORMATION**

**Patient-Reported Outcomes of Palatopharyngeal Surgery in Obstructive Sleep Apnea: A Meta-Analysis**

**Authors**: Samuel Tschopp, MD^1^; Flora Meinert, BSc^1^; Georgios Mantokoudis, MD EMBA^1^; Marco Caversaccio, MD^1^; Urs Borner, MD^1^

**Affiliations**

1 Department of Otorhinolaryngology, Head and Neck Surgery, Inselspital, University Hospital and University of Bern, Bern, Switzerland

**Corresponding Author**

Dr. med. Samuel Tschopp

Department of Otorhinolaryngology, Head and Neck Surgery

Inselspital, University Hospital, and University of Bern

Freiburgstrasse 20

3010 Bern, Switzerland

samuel.tschopp@insel.ch

ORCID: 0000-0002-5666-2092

**Table of Contents**

**Supplemental Figure S1.** Forest Plot for Epworth Sleepiness Scale Reduction of the Placebo group. The reduction was defined as the absolute difference between pre- and postoperative measurements.

**Supplemental Figure S2.** Forest Plot for Snoring Reduction of the Placebo Group. The reduction was defined as the absolute difference between pre- and postoperative measurements.

**Supplemental Figure S3.** Risk of Bias Summary Plot

**Supplemental Figure S4.** Risk of Bias Traffic Light Plot

**Supplemental Figure S5.** Contour-enhanced Funnel Plot for the Epworth Sleepiness Scale Reduction of the Included Studies

**Supplemental Figure S6.** Contour-enhanced Funnel Plot for the Snoring Reduction of the Included Studies

**Supplemental Table S1.** Abstract Review Coding Schema with the predefined Inclusion and Exclusion Criteria

**Supplemental Table S2.** Full-text Article Review Coding Schema with the predefined Inclusion and Exclusion Criteria

**Supplemental Table S3.** Risk of Bias Assessment using the ROBINS-I tool

**Supplemental Table S4.** Summary of Studies Included in the Epworth Sleepiness Scale Reduction Meta-analysis

**Supplemental Table S5.** Summary of Placebo Studies for the Epworth Sleepiness Scale Reduction

**Supplemental Table S6.** Summary of Studies Included in the Snoring Reduction Meta-analysis

**Supplemental Table S7.** Summary of Placebo Studies for the Snoring Reduction

**Supplemental Methods.** Systematic Search Syntax for all Databases

**Supplemental Figure S1.** Forest Plot for Epworth Sleepiness Scale Reduction of the Placebo group. The reduction was defined as the absolute difference between pre- and postoperative measurements.

**Supplemental Figure S2.** Forest Plot for Snoring Reduction of the Placebo Group. The reduction was defined as the absolute difference between pre- and postoperative measurements.

**Supplemental Figure 3.** Risk of Bias Summary Plot

**Supplemental Figure S4.** Risk of Bias Traffic Light Plot

**Supplemental Figure S5.** Contour-enhanced Funnel Plot for the Epworth Sleepiness Scale Reduction of the Included Studies

**Supplemental Figure S6.** Contour-enhanced Funnel Plot for the Snoring Reduction of the Included Studies

**Supplemental Table S1.** Abstract Review Coding Schema with the predefined Inclusion and Exclusion Criteria

| **Inclusion Criteria** | **Exclusion Criteria** |
| --- | --- |
| Original data | No original data presented (Reviews, guidelines, study protocols, conference proceedings, comments, posters, how-I-do-it, Letter to the Editor) |
| Outcome measures of interest (apnea-hypopnea index, daytime sleepiness or snoring) | No endpoint of interest reported in the abstract or reasonably expected in full-text manuscript |
| Adult patients | Participants <18 years old |
| Patients with sleep-disordered breathing | Patients without sleep-disordered breathing |
| Palatopharyngeal surgery (all types, including tonsillectomy) | Exclusively reporting multilevel or combined procedures |
| Surgical technique reported, implied or expected in full-text article | No surgical technique was reported or reasonably expected in full-text article |

**Supplemental Table S2.** Full-text Article Review Coding Schema with the predefined Inclusion and Exclusion Criteria

| **Domain** | **Exclusion Criteria** |
| --- | --- |
| Pharyngeal surgery without tonsillectomy | Tonsillectomy as part of pharyngeal surgery |
| Intervention | - Combined - Multilevel - Unstandardized surgeries |
| Study design | - No original investigation - No original data presented |
| Patient population | - Participants <18 years old - Preselected patients - Patients without sleep-disordered breathing |
| Multiple publication of same cohort | Overlapping patient cohort with other included study |
| Outcome | - No outcome measures of interest (apnea-hypopnea index, daytime sleepiness or snoring) - Outcome not extractable |

**Supplemental Table S3.** Risk of Bias Assessment using the ROBINS-I tool

| **Domains** | **Signaling questions** |
| --- | --- |
| **Pre-intervention** |  |
| Confounding | Is there potential for confounding of the effect of the intervention? Did the authors control for any post- intervention variables that could have been affected by the intervention? Did the authors use appropriate an analysis methods to control for any post-intervention variables (such as weight loss)? If concomitant surgeries or other intervention have been performed, the study will be excluded from the analysis. |
| Selection bias | Was selection of participants into the study based on participant characteristics observed after the start of intervention? Were adjustment techniques used that are likely to correct for the presence of selection biases? If selection criteria beyond preoperative sleep testing were used, we will consider the study of severe to critical risk of bias. |
| **Intervention** |  |
| Bias in measurement classification of Intervention | Was the surgical technique clearly described? If the surgical intervention is not described, the study will be excluded from the analysis. If there is ambiguity or inconsistent application of surgical techniques, the study will be considered of critical risk of bias. |
| **Post-intervention** |  |
| Bias due to deviations from intended intervention | Was the surgical technique standardized and the same for all patients? If the surgeons deviated from the technique due to intaoperative findings, we will consider the study of critical risk of bias. Was the intervention implemented successfully for most participants? |
| Bias due to missing data | Were outcome data available for all, or nearly all, participants? Is there evidence that results are robust to the presence of missing data? Studies with >25% lost-to-follow up will be classified as severe or critical risk of bias. |
| Bias in measurment of outcomes | Is the outcome measured using a validated sleep testing method? Is the same sleep testing used for pre- and postoperative assessment? Were any systematic errors in measurement of the outcome? |
| Bias in selection of the reported results | Is the reported effect estimate likely to be selected, on the basis of the results from multiple outcome measurements or analyses? Studies reporting only selected outcomes will be considered of severe to critical risk of bias. |

**Supplemental Information Table S4.** Summary of Studies Included in the Epworth Sleepiness Scale Reduction Meta-analysis

| Source | Surgerical Technique | Patients, No. | Gender, No. (%), male | Age, mean (SD), years | BMI, mean (SD), years | Follow-up duration, days | ESS preoperative, mean (SD) | ESS postoperative, mean (SD) | ESS reduction, mean (SD) |
| --- | --- | --- | --- | --- | --- | --- | --- | --- | --- |
| Amali et al, 2017; Iran^1^ | Radiofrequency | 20 | 85% (17/20) | 39 (10) | 28.2 (4.1) | 180 | 13.4 (6.0) | 7.7 (1.7) | 5.7 (5.4) |
| Bäck et al, 2009; Finland^2^ | Radiofrequency | 17 | 100% (17/17) | NA | 29.0 (NA) | 120 | 11.0 (5.0) | 8.5 (5.6) | 2.5 (5.3) |
| Balsevicius et al, 2015; Lithuania^3^ | Radiofrequency | 28 | 64% (18/28) | 43 (12) | 28.0 (3.7) | 120 | 8.5 (4.1) | 6.7 (3.7) | 1.8 (3.9) |
| Boudewyns et al, 2000; Belgium^4^ | Radiofrequency | 37 | 86% (32/37) | 44 (11) | 26.6 (3.2) | 160 | 8.5 (5.0) | 6.0 (4.3) | 2.5 (4.7) |
| Chiesa Estomba et al, 2015; Spain^5^ | Radiofrequency | 27 | 82% (22/27) | 49 (9) | 27.1 (2.5) | 180 | 8.8 (3.1) | 6.9 (1.7) | 1.8 (2.7) |
| Chiu et al, 2021; Taiwan^6^ | Cold Steel | 22 | 68% (15/22) | 46 (NA) | NA | 90 | 10.2 (6.3) | 5.2 (3.2) | 5.0 (5.5) |
| Choi et al, 2015; Korea^7^ | Palatal Implant | 29 | 93% (27/29) | 44 (12) | 22.3 (1.7) | 150 | 9.1 (3.4) | 7.1 (3.7) | 2.0 (3.6) |
| Coleman et al, 2000; USA^8^ | Radiofrequency | 12 | 75% (9/12) | 54 (10) | 26.6 (3.4) | 110 | 10.8 (4.4) | 8.3 (4.1) | 2.5 (4.3) |
| De Kermadec et al, 2014; France^9^ | Radiofrequency | 58 | NA | 59 (11) | NA | 2190 | 7.8 (4.6) | 7.2 (4.7) | 0.6 (4.7) |
| El-Ahl et al, 2016; Egypt^10^ | Muscle Relocation | 24 | 62% (15/24) | 35 (3) | 25.3 (1.4) | 270 | 11.7 (2.9) | 5.1 (2.2) | 6.6 (2.6) |
| Emery et al, 2000; USA^11^ | Radiofrequency | 10 | 84% (8/10) | 44 (8) | 28.5 (3.1) | 180 | 10.2 (6.1) | 6.1 (4.7) | 4.1 (5.5) |
| Emery et al, 2000; USA^11^ | Radiofrequency | 13 | 74% (10/13) | 44 (11) | 27.7 (3.8) | 100 | 8.8 (4.4) | 5.3 (3.2) | 3.5 (3.9) |
| Ferguson et al, 2003; Canada^12^ | Laser | 21 | NA | NA | NA | 240 | 10.7 (3.7) | 9.3 (3.8) | 1.4 (3.8) |
| Friedman et al, 2008; USA^13^ | Palatal Implant | 29 | 62% (18/29) | 48 (11) | 29.3 (1.9) | 90 | 12.7 (2.7) | 10.2 (3.1) | 2.4 (2.2) |
| Goessler et al, 2007; Germany^14^ | Palatal Implant | 16 | 88% (14/16) | 51 (NA) | 26.6 (NA) | 90 | 7.2 (2.5) | 4.6 (3.2) | 2.6 (2.9) |
| Haytoglu et al, 2014; Turkey^15^ | Palatal Implant | 59 | 48% (28/59) | 47 (9) | 23.7 (2.5) | 365 | 8.0 (3.5) | 7.4 (3.3) | 0.5 (3.4) |
| Haytoglu et al, 2014; Turkey^15^ | Cold Steel | 58 | 64% (37/58) | 44 (9) | 24.4 (2.5) | 365 | 8.6 (3.0) | 6.9 (3.2) | 1.7 (3.1) |
| Haytoglu et al, 2018; Turkey^16^ | Cold Steel | 22 | 54% (12/22) | 39 (5) | 28.0 (1.6) | 180 | 13.6 (3.3) | 6.4 (3.3) | 7.2 (3.3) |
| Haytoglu et al, 2018; Turkey^16^ | Cold Steel | 23 | 61% (14/23) | 41 (5) | 27.3 (1.8) | 180 | 10.8 (3.3) | 5.4 (4.8) | 5.4 (4.3) |
| Holmlund et al, 2014; Sweden^17^ | Radiofrequency | 19 | 100% (19/19) | 48 (8) | 26.5 (2.2) | 390 | 9.8 (3.6) | 8.1 (4.2) | 1.8 (3.9) |
| Hu et al, 2018; Taiwan^18^ | Palatal Implant | 10 | 100% (10/10) | 53 (12) | 26.6 (2.8) | 990 | 8.0 (6.0) | 4.8 (6.2) | 3.2 (6.1) |
| Huang et al, 2008; Taiwan^19^ | Powered Instrument | 50 | 88% (44/50) | 43 (NA) | 27.8 (3.3) | 180 | 9.8 (3.7) | 5.2 (2.2) | 4.6 (3.2) |
| Hukins et al, 2000; Austria^20^ | Radiofrequency | 20 | 80% (16/20) | 43 (11) | 27.1 (2.8) | 80 | 8.1 (4.0) | 6.7 (4.0) | 1.4 (4.0) |
| Kwon et al, 2016; Korea^21^ | Suture | 35 | 63% (22/35) | 34 (14) | 28.5 (5.6) | 90 | 7.9 (2.3) | 5.1 (3.0) | 2.9 (2.7) |
| Larrosa et al, 2004; Spain^22^ | Laser | 13 | 100% (13/13) | NA | 27.2 (1.9) | 90 | 10.4 (3.8) | 9.6 (3.8) | 0.8 (3.8) |
| Li et al, 2000; USA | Radiofrequency | 22 | 82% (18/22) | 45 (9) | 27.4 (3.7) | 420 | 8.4 (4.5) | 6.0 (4.4) | 2.4 (4.5) |
| Macdonald et al, 2006; UK^23^ | Laser | 55 | 82% (45/55) | 43 (8) | 26.8 (3.2) | 180 | 7.9 (4.2) | 6.2 (3.4) | 1.7 (3.7) |
| Mantovani et al, 2017; Italy^24^ | Suture | 19 | 95% (18/19) | 44 (9) | 26.2 (2.7) | 180 | 11.3 (5.5) | 3.9 (4.0) | 7.4 (6.3) |
| Maurer et al, 2005; Germany^25^ | Palatal Implant | 40 | NA | 42 (9) | 25.2 (2.5) | 365 | 6.1 (3.2) | 4.9 (3.1) | 1.2 (3.2) |
| Maurer et al, 2012; Germany^26^ | Palatal Implant | 10 | NA | 49 (11) | 28.6 (2.4) | 90 | 7.1 (3.3) | 6.9 (2.0) | 1.0 (3.3) |
| Moffa et al, 2024; Italy^27^ | Suture | 27 | 85% (23/27) | 56 (6) | NA | 180 | 11.6 (4.6) | 5.1 (1.5) | 6.5 (4.1) |
| Mora et al, 2012; Italy^28^ | Powered Instrument | 21 | 67% (14/21) | 47 (NA) | 31.0 (NA) | 180 | 14.0 (3.7) | 4.7 (2.2) | 9.3 (3.2) |
| Murphey AW et al, 2016; USA^29^ | Suture | 20 | 65% (13/20) | 49 (9) | 25.8 (2.7) | 90 | 6.6 (3.5) | 6.1 (3.4) | 0.5 (3.5) |
| Neruntarat et al, 2003; Thailand^30^ | Cold Steel | 56 | 89% (50/56) | 48 (10) | 25.5 (2.4) | 28 | 8.1 (3.5) | 5.1 (3.2) | 3.0 (3.4) |
| Neruntarat et al, 2011; Thailand^31^ | Palatal Implant | 92 | 88% (81/92) | 38 (10) | 28.9 (4.8) | 870 | 12.3 (2.6) | 7.9 (1.8) | 4.4 (2.3) |
| Nordgard et al, 2007; Norway^32^ | Palatal Implant | 26 | 69% (18/26) | 53 (9) | 27.2 (2.3) | 435 | 8.3 (4.7) | 5.4 (4.0) | 2.9 (4.4) |
| Rashwan et al, 2024; Egypt^33^ | Suture | 27 | NA | NA | 33.1 (2.1) | NA | 13.2 (4.7) | 6.1 (2.1) | 7.2 (4.0) |
| Rashwan et al, 2024; Egypt^33^ | Suture | 15 | NA | NA | 33.1 (2.1) | NA | 14.9 (3.7) | 7.9 (3.0) | 7.0 (3.4) |
| Server et al, 2016; Turkey^34^ | Palatal Implant | 27 | 59% (16/27) | 46 (7) | 26.2 (2.6) | 2190 | 5.5 (4.1) | 3.7 (3.9) | 1.8 (4.0) |
| Sezen et al, 2009; Turkey^35^ | Palatal Implant | 17 | 59% (10/17) | 49 (8) | 28.4 (4.3) | 90 | 6.8 (4.9) | 4.5 (2.9) | 2.3 (4.3) |
| Sher et al, 2001; USA^36^ | Radiofrequency | 105 | 80% (84/105) | 46 (10) | 27.6 (3.6) | 60 | 9.1 (4.9) | 5.9 (4.3) | 3.2 (4.6) |
| Steward et al, 2008; USA^37^ | Palatal Implant | 50 | 78% (39/50) | 47 (NA) | 27.4 (NA) | 90 | 10.6 (NA) | 8.8 (NA) | 1.8 (1.3) |
| Tatla et al, 2003; UK^38^ | Radiofrequency | 10 | 70% (7/10) | 43 (11) | 28.1 (3.0) | 180 | 9.5 (3.3) | 8.0 (4.1) | 1.5 (3.7) |
| Terris et al, 2002; USA^39^ | Radiofrequency | 10 | NA | 53 (12) | 26.7 (2.4) | 365 | 7.8 (4.7) | 6.4 (4.0) | 1.4 (4.4) |
| Tschopp et al, 2025; Switzerland^40^ | Palatal Implant | 24 | 100% (24/24) | 57 (10) | 25.7 (2.2) | 1825 | 9.6 (4.5) | 6.0 (1.0) | 3.0 (0.6) |
| Tvinnereim et al, 2007; Norway^41^ | Radiofrequency | 40 | 70% (28/40) | 46 (12) | 25.5 (7.2) | 270 | 10.5 (2.7) | 4.6 (1.6) | 5.9 (2.4) |
| Ugur et al, 2014; Turkey^42^ | Muscle Relocation | 42 | 69% (29/42) | 39 (8) | 28.5 (NA) | 720 | 11.5 (5.0) | 8.3 (3.5) | 3.2 (4.4) |
| Walker et al, 2006; USA^43^ | Palatal Implant | 53 | 83% (44/53) | 50 (12) | 28.4 (2.9) | 90 | 11.0 (5.1) | 6.9 (4.5) | 3.8 (4.8) |
| Wassmuth et al, 2000; USA^44^ | Radiofrequency | 25 | 80% (20/25) | 42 (NA) | 28.6 (3.0) | 90 | 12.4 (5.6) | 8.8 (4.6) | 3.6 (5.2) |

**Supplemental Information Table S5.** Summary of Placebo Studies for the Epworth Sleepiness Scale Reduction

| Source | Patients, No. | Gender, No. (%), male | Age, mean (SD), years | BMI, mean (SD), years | Follow-up duration, days | ESS preoperative, mean (SD) | ESS postoperative, mean (SD) | ESS reduction, mean (SD) |
| --- | --- | --- | --- | --- | --- | --- | --- | --- |
| Bäck et al, 2009; Finland^2^ | 15 | 100% (15/15) | NA | 25.6 (NA) | 120 | 8.8 (3.7) | 6.8 (3.7) | 2.0 (3.7) |
| Ferguson et al, 2003; Canada^12^ | 24 | NA | NA | NA | 460 | 10.0 (5.2) | 10.8 (9.3) | -0.8 (8.1) |
| Friedman et al, 2008; USA^13^ | 26 | 58% (15/26) | 39 (10) | 28.7 (2.3) | 90 | 11.7 (2.7) | 11.1 (2.7) | 0.5 (1.5) |
| Holmlund et al, 2014; Sweden^17^ | 13 | 100% (13/13) | 50 (8) | 26.2 (1.7) | 390 | 10.2 (5.3) | 8.3 (5.1) | 1.9 (5.2) |
| Larrosa et al, 2004; Spain^22^ | 12 | 100% (12/12) | NA | 27.0 (3.8) | 90 | 10.9 (5.6) | 10.5 (5.4) | 0.4 (5.5) |
| Maurer et al, 2012; Germany^26^ | 10 | NA | 53 (14) | 28.1 (3.0) | 90 | 7.4 (4.0) | 6.2 (3.6) | 1.8 (2.9) |
| Steward et al, 2008; USA^37^ | 50 | 80% (40/50) | 51 (NA) | 27.8 (NA) | 90 | 10.7 (NA) | 9.2 (NA) | 1.5 (1.0) |

**Supplemental Table S6.** Summary of Studies Included in the Snoring Reduction Meta-analysis

| Source | Surgerical Technique | Patients, No. | Gender, No. (%), male | Age, mean (SD), years | BMI, mean (SD), years | Follow-up duration, days | Snoring preoperative, mean (SD) | Snoring postoperative, mean (SD) | Snoring reduction, mean (SD) |
| --- | --- | --- | --- | --- | --- | --- | --- | --- | --- |
| Al Bassiouny et al, 2009; Kuwait^45^ | Muscle Relocation | 32 | 81% (26/32) | 43 (NA) | 28.2 (NA) | 90 | 8.9 (0.9) | 2.3 (0.4) | 6.6 (0.8) |
| Bäck et al, 2009; Finland^2^ | Radiofrequency | 17 | 100% (17/17) | NA | 29.0 (NA) | 120 | 6.2 (1.9) | 4.8 (1.9) | 1.5 (1.9) |
| Balsevicius et al, 2015; Lithuania^3^ | Radiofrequency | 28 | 64% (18/28) | 43 (12) | 28.0 (3.7) | 120 | 6.9 (1.9) | 3.2 (2.0) | 3.7 (2.0) |
| Boudewyns et al, 2000; Belgium^4^ | Radiofrequency | 37 | 86% (32/37) | 44 (11) | 26.6 (3.2) | 160 | 7.6 (2.1) | 4.0 (NA) | 3.6 (2.7) |
| Chiesa Estomba et al, 2015; Spain^5^ | Radiofrequency | 27 | 82% (22/27) | 49 (9) | 27.1 (2.5) | 365 | 8.1 (0.9) | 4.9 (0.8) | 3.2 (0.9) |
| Choi et al, 2015; Korea^7^ | Palatal Implant | 29 | 93% (27/29) | 44 (12) | 22.3 (1.7) | 150 | 6.3 (1.4) | 3.0 (1.5) | 3.3 (1.5) |
| Coleman et al, 2000; USA^8^ | Radiofrequency | 12 | 75% (9/12) | 54 (10) | 26.6 (3.4) | 110 | 8.3 (2.1) | 2.1 (1.4) | 6.2 (1.9) |
| Daneshi et al, 2020; Iran^46^ | Palatal Implant | 13 | 85% (11/13) | 49 (12) | 28.3 (3.6) | 365 | 7.6 (0.6) | 3.5 (0.5) | 4.1 (0.6) |
| De Kermadec et al, 2014; France^9^ | Radiofrequency | 58 | NA | 59 (11) | NA | 2190 | 8.1 (1.5) | 5.7 (2.9) | 2.4 (2.5) |
| El-Ahl et al, 2016; Egypt^10^ | Muscle Relocation | 24 | 62% (15/24) | 35 (3) | 25.3 (1.4) | 270 | 8.2 (1.4) | 2.1 (1.4) | 6.1 (1.4) |
| Elbassiouny et al, 2015; Egypt^47^ | Muscle Relocation | 28 | 82% (23/28) | 41 (5) | 29.4 (3.8) | 180 | 8.6 (3.7) | 2.3 (2.7) | 6.3 (3.3) |
| Elbassiuony et al, 2012; Kuwait^48^ | Muscle Relocation | 93 | 93% (86/93) | 43 (6) | 28.2 (2.9) | 180 | 8.8 (1.5) | 2.9 (2.5) | 5.9 (2.2) |
| Emery et al, 2000; USA^11^ | Radiofrequency | 10 | 84% (8/10) | 44 (8) | 28.5 (3.1) | 180 | 7.8 (1.8) | 2.3 (2.1) | 5.5 (2.0) |
| Emery et al, 2000; USA^11^ | Radiofrequency | 13 | 74% (10/13) | 44 (11) | 27.7 (3.8) | 100 | 8.9 (1.7) | 2.5 (0.8) | 6.4 (1.5) |
| Ferguson et al, 2003; Canada^12^ | Laser | 21 | NA | NA | NA | 240 | 9.2 (1.0) | 4.8 (2.8) | 4.4 (2.5) |
| Friedman et al, 2008; USA^13^ | Palatal Implant | 29 | 62% (18/29) | 48 (11) | 29.3 (1.9) | 90 | 9.1 (1.5) | 4.4 (2.4) | 4.7 (2.1) |
| Goessler et al, 2007; Germany^14^ | Palatal Implant | 16 | 88% (14/16) | 51 (NA) | 26.6 (NA) | 90 | 8.3 (1.8) | 4.7 (2.5) | 3.6 (2.2) |
| Goktas et al, 2014; Germany^49^ | Laser | 25 | 88% (22/25) | 63 (NA) | 30.0 (4.4) | 4020 | 9.5 (0.9) | 5.3 (3.3) | 4.2 (3.0) |
| Haytoglu et al, 2018; Turkey^16^ | Cold Steel | 22 | 54% (12/22) | 39 (5) | 28.0 (1.6) | 180 | 8.1 (1.7) | 3.0 (2.7) | 5.1 (2.4) |
| Haytoglu et al, 2018; Turkey^16^ | Cold Steel | 23 | 61% (14/23) | 41 (5) | 27.3 (1.8) | 180 | 8.6 (1.4) | 2.8 (2.9) | 5.8 (2.5) |
| Hu et al, 2018; Taiwan^18^ | Palatal Implant | 10 | 100% (10/10) | 53 (12) | 26.6 (2.8) | 990 | 8.4 (1.3) | 4.0 (0.9) | 4.4 (1.1) |
| Huang et al, 2008; Taiwan^19^ | Powered Instrument | 50 | 88% (44/50) | 43 (NA) | 27.8 (3.3) | 180 | 7.5 (2.0) | 2.4 (1.3) | 5.1 (1.8) |
| Hukins et al, 2000; Austria^20^ | Radiofrequency | 20 | 80% (16/20) | 43 (11) | 27.1 (2.8) | 80 | 7.5 (1.5) | 4.6 (2.5) | 2.9 (2.2) |
| Kwon et al, 2016; Korea^21^ | Suture | 35 | 63% (22/35) | 34 (14) | 28.5 (5.6) | 90 | 8.7 (2.3) | 4.1 (1.7) | 4.6 (2.0) |
| Lee et al, 2014; Taiwan^50^ | Palatal Implant | 14 | 100% (14/14) | 37 (2) | 24.2 (0.5) | 90 | 7.7 (0.4) | 4.4 (0.6) | 3.3 (0.5) |
| Lee et al, 2014; Taiwan^50^ | Radiofrequency | 14 | 80% (11/14) | 35 (2) | 23.6 (0.7) | 90 | 6.6 (0.5) | 5.2 (0.4) | 1.4 (0.5) |
| Li et al, 2000; USA^51^ | Radiofrequency | 22 | 82% (18/22) | 45 (9) | 27.4 (3.7) | 420 | 8.3 (1.8) | 3.8 (2.5) | 4.5 (2.3) |
| Mantovani et al, 2017; Italy^24^ | Suture | 19 | 95% (18/19) | 44 (9) | 26.2 (2.7) | 180 | 9.5 (0.7) | 2.1 (1.7) | 7.8 (1.5) |
| Maurer et al, 2005; Germany^25^ | Palatal Implant | 40 | NA | 42 (9) | 25.2 (2.5) | 365 | NA (NA) | NA (NA) | 2.2 (2.5) |
| Maurer et al, 2012; Germany^26^ | Palatal Implant | 10 | NA | 49 (11) | 28.6 (2.4) | 90 | 7.1 (1.3) | 5.5 (2.3) | 2.7 (1.7) |
| Mora et al, 2012; Italy^28^ | Powered Instrument | 21 | 67% (14/21) | 47 (NA) | 31.0 (NA) | 180 | 7.9 (0.8) | 1.6 (0.3) | 6.3 (0.7) |
| Murphey AW et al, 2016; USA^29^ | Suture | 20 | 65% (13/20) | 49 (9) | 25.8 (2.7) | 90 | 5.9 (2.1) | 4.2 (2.9) | 1.8 (2.6) |
| Neruntarat et al, 2003; Thailand^30^ | Cold Steel | 56 | 89% (50/56) | 48 (10) | 25.5 (2.4) | 28 | 8.2 (3.4) | 2.6 (1.4) | 5.6 (3.0) |
| Neruntarat et al, 2011; Thailand^31^ | Palatal Implant | 92 | 88% (81/92) | 38 (10) | 28.9 (4.8) | 870 | 8.2 (1.2) | 3.8 (2.3) | 4.4 (2.0) |
| Nordgard et al, 2007; Norway^32^ | Palatal Implant | 26 | 69% (18/26) | 53 (9) | 27.2 (2.3) | 435 | 8.4 (1.4) | 4.6 (2.5) | 3.8 (2.2) |
| Nordgard et al, 2006; Norway^52^ | Palatal Implant | 30 | NA | 44 (44) | 26.1 (2.3) | 365 | 7.1 (2.1) | 4.8 (3.1) | 2.3 (2.7) |
| Picavet et al, 2023; Germany^53^ | Laser | 19 | 65% (12/19) | 43 (10) | 26.6 (3.6) | 90 | 7.9 (2.0) | 4.7 (2.8) | 3.2 (2.5) |
| Rotenberg et al, 2012; Canada^54^ | Palatal Implant | 23 | 87% (20/23) | 55 (22) | 27.6 (5.5) | 1460 | 9.5 (0.6) | 7.0 (1.8) | 2.5 (1.6) |
| Server et al, 2016; Turkey^34^ | Palatal Implant | 27 | 59% (16/27) | 46 (7) | 26.2 (2.6) | 2190 | 7.3 (2.3) | 6.0 (3.0) | 1.3 (2.7) |
| Sezen et al, 2009; Turkey^35^ | Palatal Implant | 17 | 59% (10/17) | 49 (8) | 28.4 (4.3) | 90 | 7.3 (1.0) | 4.7 (1.9) | 2.6 (1.6) |
| Sher et al, 2001; USA^36^ | Radiofrequency | 105 | 80% (84/105) | 46 (10) | 27.6 (3.6) | 60 | 7.8 (2.1) | 3.2 (2.2) | 4.6 (2.2) |
| Stuck et al, 2009; Germany^55^ | Radiofrequency | 19 | NA | NA | 25.5 (2.8) | 600 | 8.6 (1.5) | 2.0 (2.1) | 6.6 (1.9) |
| Tatla et al, 2003; UK^38^ | Radiofrequency | 10 | 70% (7/10) | 43 (11) | 28.1 (3.0) | 180 | 7.7 (1.1) | 4.6 (1.9) | 3.1 (2.0) |
| Terris et al, 2002; USA^39^ | Radiofrequency | 10 | NA | 53 (12) | 26.7 (2.4) | 365 | 7.5 (2.1) | 3.1 (2.6) | 4.4 (2.4) |
| Tschopp et al, 2025; Switzerland^40^ | Palatal Implant | 24 | 100% (24/24) | 57 (10) | 25.7 (2.2) | 1825 | 8.4 (1.0) | 6.0 (2.1) | 2.0 (0.5) |
| Tvinnereim et al, 2007; Norway^41^ | Radiofrequency | 40 | 70% (28/40) | 46 (12) | 25.5 (7.2) | 270 | 8.1 (0.9) | 2.8 (1.0) | 5.3 (0.9) |
| Ugur et al, 2014; Turkey^42^ | Muscle Relocation | 42 | 69% (29/42) | 39 (8) | 28.5 (NA) | 720 | 6.2 (8.3) | 3.4 (7.6) | 2.8 (8.0) |
| Walker et al, 2006; USA^43^ | Palatal Implant | 53 | 83% (44/53) | 50 (12) | 28.4 (2.9) | 90 | 7.9 (2.1) | 4.0 (3.0) | 4.0 (2.9) |

**Supplemental Table S7.** Summary of Placebo Studies for the Snoring Reduction

| Source | Patients, No. | Gender, No. (%), male | Age, mean (SD), years | BMI, mean (SD), years | Follow-up duration, days | Snoring preoperative, mean (SD) | Snoring postoperative, mean (SD) | Snoring reduction, mean (SD) |
| --- | --- | --- | --- | --- | --- | --- | --- | --- |
| Bäck et al, 2009; Finland^2^ | 15 | 100% (15/15) | NA | 25.6 (NA) | 120 | 6.0 (2.3) | 5.8 (2.0) | 0.2 (2.2) |
| Ferguson et al, 2003; Canada^12^ | 24 | NA | NA | NA | 460 | 8.9 (1.1) | 8.5 (1.2) | 0.4 (1.2) |
| Friedman et al, 2008; USA^13^ | 26 | 58% (15/26) | 39 (10) | 28.7 (2.3) | 90 | 9.3 (0.7) | 8.4 (1.0) | 0.7 (0.9) |
| Maurer et al, 2012; Germany^26^ | 10 | NA | 53 (14) | 28.1 (3.0) | 90 | 7.7 (1.8) | 5.1 (2.8) | 2.1 (3.1) |
| Picavet et al, 2023; Germany^53^ | 15 | 75% (11/15) | 44 (8) | 26.9 (3.9) | 90 | 8.1 (1.7) | 8.0 (1.6) | 0.1 (1.7) |

**Supplemental Methods.** Systematic Search Syntax for all Databases

**Information Sources and Content Coverage Dates**

- MEDLINE (R) ALL (Ovid), Coverage 1946-Present
- Embase (Ovid), Coverage 1974-Present
- Cochrane Library (Wiley), Coverage, 1996-Present
- Clinical Trial registers
- WHO International Clinical Trials Registry Platform (ICTRP)
- Web of Science Core Collection, Coverage 1637-Present
- CINAHL® with Full Text, Coverage 1981-Present

**Search strategy & deduplication process**

The literature search was performed by combining controlled vocabulary terms (e.g., MeSH, EMTREE) and free-text terms for the following concepts: apnea, various types of soft palate surgery (including uvulopalatopharyngoplasty), implants, and outcomes such as apnea–hypopnea index, sleepiness, and snoring. Filters for human studies and children were implemented in Web of Science, CINAHL, Medline, and Embase. Search strategies were translated accordingly for each information source. A total of 7028 records were retrieved. 2638 duplicates were removed with Deduklick, a fully automated deduplication software. Additionally, 6 additional records were removed from the database due to duplication identified and evaluated in Covidence. A new total of 4390 records were prepared in Covidence for screening.

Reference: Borissov N, Haas Q, Minder B, Kopp-Heim D, von Gernler M, Janka H, Teodoro D, Amini P. Reducing systematic review burden using Deduklick: a novel, automated, reliable, and explainable deduplication algorithm to foster medical research. Systematic reviews. 2022 Dec;11(1):1-0. <https://pubmed.ncbi.nlm.nih.gov/35978441/>

**MEDLINE (R) ALL (Ovid)**

1	Sleep Apnea Syndromes/ or exp Sleep Apnea, Obstructive/ or apnea/ or Airway Resistance/

2	(((apn?e* or hypopn?e*) adj2 (sleep* or obstructive or syndrome* or nocturnal)) or OSA or OSAS or OSAHS).ti,ab.

3	((sleep* adj3 breath*) or ((airway or air-way) adj2 (resistanc* or obstruction* or blockage or blocked))).ti,ab.

4	or/2-3

5	or/1,4

6	otorhinolaryngologic surgical procedures/ or exp "Prostheses and Implants"/

7	exp Palate, Soft/su [Surgery]

8	Pharynx/su [Surgery]

9	or/6-8

10	(uvulopalatopharyngoplast* or uvulo-palatopharyngoplast* or palatopharyngoplast* or uvulopalato-pharyngoplast* or uvulo-palato-pharyngoplast* or palatouvulopharyngoplast* or pharyngouvulopalatoplast* or uvulopharyngopalatoplast* or uvulopalatoplast* or uvulo-palatoplast* or uvulopharyngoplast* or uvulo-pharyngoplast* or palatoplast* or pharyngoplast* or velopharyngoplast* or velo-pharyngoplast* or Han-uvulopalatopharyngoplast* or sphincterpharyngoplast* or sphincteroplasty or palato-uvulopharyngoplast* or pharyngo-uvulopalatoplast* or uvulo-pharyngopalatoplast* or palatouvulo-pharyngoplast* or pharyngouvulo-palatoplast* or uvulopharyngo-palatoplast* or palato-uvulo-pharyngoplast* or pharyngo-uvulo-palatoplast* or uvulo-pharyngo-palatoplast* or pharyngopalatoplast* or pharyngo-palatoplast* or UPPP or HUPPP or UP3 or LAUP or UVPP or ((palat* or pillar* or velopharyng* or pharyng* or pharynx) adj2 (implant* or prosthese*))).ti,ab.

11	or/9-10

12	apnea hypopnea index/ or exp sleep disorder assessment/ or epworth sleepiness scale/ or snoring/ or exp daytime somnolence/

13	(((apn?e* or hypopn?e* or respirator*) adj2 index*) or AHI or snoring* or snort* or gasp* or (epworth adj2 scale) or sleepiness or somnolence or drowsiness or polysomnograph* or somnograph*).ti,ab.

14	or/12-13

15	and/5,11,14

16	(exp animals/ or exp animal experimentation/ or exp models, animal/ or exp plants/ or exp fungi/) not humans/

17	15 not 16

18	(child* or stepchild* or step-child* or kid or kids or girl or girls or boy or boys or teen* or youth* or youngster* or adolescent* or adolescence or preschool* or pre-school* or kindergarten* or school* or juvenile* or minors or p?ediatric* or infan* or toddler* or newborn* or new-born* or PICU?).ti.

19	17 not 18

**Embase (Ovid)**

1	sleep apnea syndromes/ or mixed sleep apnea/ or obstructive sleep apnea/ or apnea/ or snoring/ or obstructive airway disease/ or airway resistance/

2	(((apn?e* or hypopn?e*) adj2 (sleep* or obstructive or syndrome* or nocturnal)) or OSA or OSAS or OSAHS).ti,ab.

3	((sleep* adj3 breath*) or ((airway or air-way) adj2 (resistanc* or obstruction* or blockage or blocked))).ti,ab.

4	or/1-3

5	exp throat surgery/ or palatoplasty/

6	soft palate/su [Surgery]

7	exp pharynx/su [Surgery]

8	exp prosthesis/ or exp implant/

9	(uvulopalatopharyngoplast* or uvulo-palatopharyngoplast* or palatopharyngoplast* or uvulopalato-pharyngoplast* or uvulo-palato-pharyngoplast* or palatouvulopharyngoplast* or pharyngouvulopalatoplast* or uvulopharyngopalatoplast* or uvulopalatoplast* or uvulo-palatoplast* or uvulopharyngoplast* or uvulo-pharyngoplast* or palatoplast* or pharyngoplast* or velopharyngoplast* or velo-pharyngoplast* or Han-uvulopalatopharyngoplast* or sphincterpharyngoplast* or sphincteroplasty or palato-uvulopharyngoplast* or pharyngo-uvulopalatoplast* or uvulo-pharyngopalatoplast* or palatouvulo-pharyngoplast* or pharyngouvulo-palatoplast* or uvulopharyngo-palatoplast* or palato-uvulo-pharyngoplast* or pharyngo-uvulo-palatoplast* or uvulo-pharyngo-palatoplast* or pharyngopalatoplast* or pharyngo-palatoplast* or UPPP or HUPPP or UP3 or LAUP or UVPP or ((palat* or pillar* or velopharyng* or pharyng* or pharynx) adj2 (implant* or prosthese*))).ti,ab.

10	or/5-9

11	apnea hypopnea index/ or exp sleep disorder assessment/ or epworth sleepiness scale/ or snoring/ or exp daytime somnolence/

12	(((apn?e* or hypopn?e* or respirator*) adj2 index*) or AHI or snoring* or snort* or gasp* or (epworth adj2 scale) or sleepiness or somnolence or drowsiness or polysomnograph* or somnograph*).ti,ab.

13	or/11-12

14	and/4,10,13

15	(exp animal/ or exp animal experiment/ or exp animal model/ or exp plant/ or exp fungus/ or (rat or rats or animal? or mouse or mice or "in vivo" or rabbit? or murine? or pig or pigs or dog or dogs or bovine? or fish or fishes or vertebrate? or cat or cats or rodent? or mammal? or chicken? or monkey? or sheep or canine? or porcine? or cattle or bird? or hamster? or primate?).ti,ab,kw.) not exp human/

16	14 not 15

17	(child* or stepchild* or step-child* or kid or kids or girl or girls or boy or boys or teen* or youth* or youngster* or adolescent* or adolescence or preschool* or pre-school* or kindergarten* or school* or juvenile* or minors or p?ediatric* or infan* or toddler* or newborn* or new-born* or PICU?).ti.

18	16 not 17

19	Conference Abstract.pt.

20	18 not 19

**Cochrane Library (Wiley)**

Advanced Search Mode

#1	(((apn?e* or hypopn?e*) NEAR/2 (sleep* or obstructive or syndrome* or nocturnal)) or OSA or OSAS or OSAHS):ti,ab,kw

#2	((sleep* NEAR/3 breath*) or ((airway or air-way) NEAR/2 (resistanc* or obstruction* or blockage or blocked))):ti,ab,kw

#3	#1 OR #2

#4	(uvulopalatopharyngoplast* or uvulo-palatopharyngoplast* or palatopharyngoplast* or uvulopalato-pharyngoplast* or uvulo-palato-pharyngoplast* or palatouvulopharyngoplast* or pharyngouvulopalatoplast* or uvulopharyngopalatoplast* or uvulopalatoplast* or uvulo-palatoplast* or uvulopharyngoplast* or uvulo-pharyngoplast* or palatoplast* or pharyngoplast* or velopharyngoplast* or velo-pharyngoplast* or Han-uvulopalatopharyngoplast* or sphincterpharyngoplast* or sphincteroplasty or palato-uvulopharyngoplast* or pharyngo-uvulopalatoplast* or uvulo-pharyngopalatoplast* or palatouvulo-pharyngoplast* or pharyngouvulo-palatoplast* or uvulopharyngo-palatoplast* or palato-uvulo-pharyngoplast* or pharyngo-uvulo-palatoplast* or uvulo-pharyngo-palatoplast* or pharyngopalatoplast* or pharyngo-palatoplast* or UPPP or HUPPP or UP3 or LAUP or UVPP or ((palat* or pillar* or velopharyng* or pharyng* or pharynx) NEAR/2 (implant* or prosthese*))):ti,ab,kw

#5	(((apn?e* or hypopn?e* or respirator* ) NEAR/2 index* ) or AHI or snoring* or snort* or gasp* or (epworth NEAR/2 scale ) or sleepiness or somnolence or drowsiness or polysomnograph* or somnograph*):ti,ab,kw

#6	#3 AND #4 AND #5

#7	(child* OR stepchild* OR step-child* OR kid OR kids OR girl OR girls OR boy OR boys OR teen* OR youth* OR youngster* OR adolescent* OR adolescence OR preschool* OR pre-school* OR kindergarten* OR school* OR juvenile* OR minors OR p?ediatric* OR infan* OR toddler* OR newborn* OR new-born* OR PICU?):ti

#8	#6 NOT #7

**Clinical Trial. gov**

Advanced Search Mode

(apnea OR apnoea OR hypopnea OR hypopnoea) AND (surgery OR uvulopalatopharyngoplasty OR palatoplasty OR pharyngoplasty OR sphincteroplasty OR UPPP OR implant* or prosthese*) AND (AHI OR index OR snoring OR Epworth OR sleepiness OR somnolence OR polysomnography)

[Note: The string related to outcomes was introduced in the field; other terms and concepts related to apnea, uvulopalatopharyngoplasty, and implants were introduced in the field condition.]

**WHO International Clinical Trials Registry Platform (ICTRP)**

Advanced Search Mode

Recruitment status = all

(apnea OR apnoea) AND (uvulopalatopharyngoplasty OR palatoplasty OR pharyngoplasty OR sphincteroplasty or implant or prostheses)

[Note: The string was introduced in the three main search fields of the basic search.]

**Web of Science Core Collection (Clarivate)**

Advanced Search Mode

1	"TS=(((apn$e* OR hypopn$e*) NEAR/2 (sleep* OR obstructive OR syndrome* OR nocturnal)) OR OSA OR OSAS OR OSAHS OR ((airway OR air-way) NEAR/2 (resistanc* OR obstruction* OR blockage OR blocked))) "

2	"TI=(((apn$e* OR hypopn$e*) NEAR/2 (sleep* OR obstructive OR syndrome* OR nocturnal)) OR OSA OR OSAS OR OSAHS OR ((airway OR air-way) NEAR/2 (resistanc* OR obstruction* OR blockage OR blocked))) "

3	"AB=(((apn$e* OR hypopn$e*) NEAR/2 (sleep* OR obstructive OR syndrome* OR nocturnal)) OR OSA OR OSAS OR OSAHS OR ((airway OR air-way) NEAR/2 (resistanc* OR obstruction* OR blockage OR blocked))) "

4	"#1 OR #2 OR #3	"

5	"TS=(uvulopalatopharyngoplast* OR palatopharyngoplast* OR palatouvulopharyngoplast* OR pharyngouvulopalatoplast* OR uvulopharyngopalatoplast* OR uvulopalatoplast* OR uvulopharyngoplast* OR palatoplast* OR pharyngoplast* OR velopharyngoplast* OR sphincterpharyngoplast* OR sphincteroplasty OR pharyngopalatoplast* OR UPPP OR HUPPP OR UP3 OR LAUP OR UVPP OR ((palat* OR pillar* OR velopharyngeal OR pharyng) NEAR/2 (implant* OR prosthese*)))"

6	"TI=(uvulopalatopharyngoplast* OR palatopharyngoplast* OR palatouvulopharyngoplast* OR pharyngouvulopalatoplast* OR uvulopharyngopalatoplast* OR uvulopalatoplast* OR uvulopharyngoplast* OR palatoplast* OR pharyngoplast* OR velopharyngoplast* OR sphincterpharyngoplast* OR sphincteroplasty OR pharyngopalatoplast* OR UPPP OR HUPPP OR UP3 OR LAUP OR UVPP OR ((palat* OR pillar* OR velopharyngeal OR pharyng) NEAR/2 (implant* OR prosthese*)))"

7	"AB=(uvulopalatopharyngoplast* OR palatopharyngoplast* OR palatouvulopharyngoplast* OR pharyngouvulopalatoplast* OR uvulopharyngopalatoplast* OR uvulopalatoplast* OR uvulopharyngoplast* OR palatoplast* OR pharyngoplast* OR velopharyngoplast* OR sphincterpharyngoplast* OR sphincteroplasty OR pharyngopalatoplast* OR UPPP OR HUPPP OR UP3 OR LAUP OR UVPP OR ((palat* OR pillar* OR velopharyngeal OR pharyng) NEAR/2 (implant* OR prosthese*)))"

8	"#5 OR #6 OR #7	"

9	"TS=(((apn$e* OR hypopn$e* OR respirator*) NEAR/2 index*) OR AHI OR snoring* OR snort* OR gasp* OR (epworth NEAR/2 scale) OR sleepiness OR somnolence OR drowsiness OR polysomnograph* OR somnograph*)"

10	"TI=(((apn$e* OR hypopn$e* OR respirator*) NEAR/2 index*) OR AHI OR snoring* OR snort* OR gasp* OR (epworth NEAR/2 scale) OR sleepiness OR somnolence OR drowsiness OR polysomnograph* OR somnograph*)"

11	"AB=(((apn$e* OR hypopn$e* OR respirator*) NEAR/2 index*) OR AHI OR snoring* OR snort* OR gasp* OR (epworth NEAR/2 scale) OR sleepiness OR somnolence OR drowsiness OR polysomnograph* OR somnograph*)"

12	"#11 OR #10 OR #9"

13	"#12 AND #8 AND #4"

14	"SU=(Veterinary Sciences)"

15	"#13 NOT #14"

16	"TI=(child* OR stepchild* OR step-child* OR kid OR kids OR girl OR girls OR boy OR boys OR teen* OR youth* OR youngster* OR adolescent* OR adolescence OR preschool* OR pre-school* OR kindergarten* OR school* OR juvenile* OR minors OR p$ediatric* OR infan* OR toddler* OR newborn* OR new-born* OR PICU$)"

17	"AB=(child* OR stepchild* OR step-child* OR kid OR kids OR girl OR girls OR boy OR boys OR teen* OR youth* OR youngster* OR adolescent* OR adolescence OR preschool* OR pre-school* OR kindergarten* OR school* OR juvenile* OR minors OR p$ediatric* OR infan* OR toddler* OR newborn* OR new-born* OR PICU$)"

18	"#17 OR #16"

19	"#15 NOT #18"

**CINAHL® with Full Text**

Advanced search Mode = Boolean/Phrase

S16	S14 NOT S15

S15	TI (child* OR stepchild* OR step-child* OR kid OR kids OR girl OR girls OR boy OR boys OR teen* OR youth* OR youngster* OR adolescent* OR adolescence OR preschool* OR pre-school* OR kindergarten* OR school* OR juvenile* OR minors OR p#ediatric* OR infan* OR toddler* OR newborn* OR new-born* OR PICU#)

S14	S5 AND S10 AND S13

S13	S11 OR S12

S12	TI (((apn#e* OR hypopn#e* OR respirator* ) N2 index* ) OR AHI OR snoring* OR snort* OR gasp* OR (epworth N2 scale ) OR sleepiness OR somnolence OR drowsiness OR polysomnograph* OR somnograph*) OR AB (((apn#e* OR hypopn#e* OR respirator* ) N2 index* ) OR AHI OR snoring* OR snort* OR gasp* OR (epworth N2 scale ) OR sleepiness OR somnolence OR drowsiness OR polysomnograph* OR somnograph*)

S11	(MH "apnea hypopnea index") OR (MH "sleep disorder assessment+") OR (MH "epworth sleepiness scale") OR (MH snoring) OR (MH "daytime somnolence+")

S10	S6 OR S7 OR S8 OR S9

S9	TI (uvulopalatopharyngoplast* OR palatopharyngoplast* OR palatouvulopharyngoplast* OR pharyngouvulopalatoplast* OR uvulopharyngopalatoplast* OR uvulopalatoplast* OR uvulopharyngoplast* OR palatoplast* OR pharyngoplast* OR velopharyngoplast* OR sphincterpharyngoplast* OR sphincteroplasty OR pharyngopalatoplast* OR UPPP OR HUPPP OR UP3 OR LAUP OR UVPP OR ((palat* OR pillar* OR velopharyngeal OR pharyng) N2 (implant* OR prosthese*))) OR AB (uvulopalatopharyngoplast* OR palatopharyngoplast* OR palatouvulopharyngoplast* OR pharyngouvulopalatoplast* OR uvulopharyngopalatoplast* OR uvulopalatoplast* OR uvulopharyngoplast* OR palatoplast* OR pharyngoplast* OR velopharyngoplast* OR sphincterpharyngoplast* OR sphincteroplasty OR pharyngopalatoplast* OR UPPP OR HUPPP OR UP3 OR LAUP OR UVPP OR ((palat* OR pillar* OR velopharyngeal OR pharyng) N2 (implant* OR prosthese*)))

S8	(MH "Pharynx+/SU")

S7	(MH "Palate, Soft+/SU")

S6	(MH "Surgery, Otorhinolaryngologic+") OR (MH "Prostheses and Implants+")

S5	S1 OR S2 OR S3 OR S4

S4	AB (OSA OR OSAS OR OSAHS)

S3	TI ((airway OR air-way) N2 (resistanc* OR obstruction* OR blockage)) OR AB ((airway OR air-way) N2 (resistanc* OR obstruction* OR blockage))

S2	TI ((apn#e* OR hypopn#e*) N2 (sleep* or obstructive or syndrome* or nocturnal or index)) OR AB ((apn#e* OR hypopn#e*) N2 (sleep* or obstructive or syndrome* or nocturnal or index))

S1	(MH "Sleep Apnea Syndromes") OR (MH "Sleep Apnea, Obstructive") OR (MH "Airway Resistance")

**References**

1.	Amali A, Motiee-Langroudi M, Saedi B, Rahavi-Ezabadi S, Karimian A, Amirzargar B. A Comparison of Uvulopalatopharyngoplasty and Modified Radiofrequency Tissue Ablation in Mild to Moderate Obstructive Sleep Apnea: A Randomized Clinical Trial. *J Clin Sleep Med*. 2017;13(09):1089-1096. doi:10.5664/jcsm.6730

2.	Bäck LJJ, Liukko T, Rantanen I, et al. Radiofrequency surgery of the soft palate in the treatment of mild obstructive sleep apnea is not effective as a single‐stage procedure: A randomized single‐blinded placebo‐controlled trial. *The Laryngoscope*. 2009;119(8):1621-1627. doi:10.1002/lary.20562

3.	Balsevicius T, Uloza V, Sakalauskas R, Miliauskas S, Jarutiene I. Efficacy of radiofrequency treatment of the soft palate for patients with mild to moderate obstructive sleep apnea hypopnea syndrome: treatment protocol with nine lesions to the soft palate. *Sleep Breath*. 2015;19(3):1003-1009. doi:10.1007/s11325-014-1004-y

4.	Boudewyns A, Van De Heyning P. Temperature-controlled radiofrequency tissue volume reduction of the soft palate (somnoplasty) in the treatment of habitual snoring: results of a European multicenter trial. *Acta Otolaryngol Stockh*. 2000;120(8):981-985.

5.	Chiesa Estomba CM, Rivera Schmitz T, Ossa Echeverri CC, Betances Reinoso FA, Farina Conde J, Alonso Parraga D. The treatment of snoring by radiofrequency-assisted uvulopalatoplasty and results after one-session protocol: a prospective, longitudinal, non-randomized study. *Eur Arch Otorhinolaryngol*. 2015;272(10):3059-3063. doi:10.1007/s00405-015-3617-6

6.	Chiu FH, Chen CY, Lee JC, Hsu YS. Effect of Modified Uvulopalatopharyngoplasty without Tonsillectomy on Obstructive Sleep Apnea: Polysomnographic Outcome and Correlation with Drug-Induced Sleep Endoscopy. *Nat Sci Sleep*. 2021;Volume 13:11-19. doi:10.2147/NSS.S286203

7.	Choi JH, Cho JH, Chung YS, Kim JW, Kim SW. Effect of the Pillar implant on snoring and mild obstructive sleep apnea: A multicenter study in Korea. *Laryngoscope*. 2015;125(5):1239-1243. doi:10.1002/lary.24975

8.	Coleman SC, Smith TL. Midline radiofrequency tissue reduction of the palate for bothersome snoring and sleep-disordered breathing: A clinical trial. *Otolaryngol Head Neck Surg*. 2000;122(3):387-394.

9.	De Kermadec H, Blumen MB, Engalenc D, Vezina JP, Chabolle F. Radiofrequency of the soft palate for sleep-disordered breathing: A 6-year follow-up study. *Eur Ann Otorhinolaryngol Head Neck Dis*. 2014;131(1):27-31. doi:10.1016/j.anorl.2013.04.005

10.	El-Ahl MA, El-Anwar MW. Expansion Pharyngoplasty by New Simple Suspension Sutures without Tonsillectomy. *Otolaryngol Head Neck Surg*. 2016;155(6):1065-1068.

11.	Emery BE, Flexon PB. Radiofrequency volumetric tissue reduction of the soft palate: A new treatment for snoring. *Laryngoscope*. 2000;110(7):1092-1098. doi:10.1097/00005537-200007000-00005

12.	Ferguson KA, Heighway K, Ruby RR. A randomized trial of laser-assisted uvulopalatoplasty in the treatment of mild obstructive sleep apnea. *Am J Respir Crit Care Med*. 2003;167(1):15-19.

13.	Friedman M, Schalch P, Lin HC, Kakodkar KA, Joseph NJ, Mazloom N. Palatal implants for the treatment of snoring and obstructive sleep apnea/hypopnea syndrome. *Otolaryngol Head Neck Surg*. 2008;138(2):209-216. doi:10.1016/j.otohns.2007.10.026

14.	Goessler UR, Hein G, Verse T, Stuck BA, Hormann K, Maurer JT. Soft palate implants as a minimally invasive treatment for mild to moderate obstructive sleep apnea. *Acta Otolaryngol Stockh*. 2007;127(5):527-531.

15.	Haytoglu S, Kayabasoglu G, Sezen OS. Comparison of two techniques in simple snoring and obstructive sleep apnea syndrome patients: Palatal implant or uvulopalatal flap. *Eur J Gen Med*. 2014;11(3):179-186. doi:10.15197/sabad.1.11.66

16.	Haytoğlu S, Arikan OK, Muluk NB, Tuhanioğlu B, Çörtük M. Comparison of Anterior Palatoplasty and Uvulopalatal Flap Placement for Treating Mild and Moderate Obstructive Sleep Apnea. *Ear Nose Throat J*. 2018;97(3):69-78. doi:10.1177/014556131809700308

17.	Holmlund T, Levring-Jaghagen E, Franklin KA, Lindkvist M, Berggren D. Effects of Radiofrequency versus sham surgery of the soft palate on daytime sleepiness. *Laryngoscope*. 2014;124(10):2422-2426. doi:10.1002/lary.24580

18.	Hu HC, Kuo CL, Tung TH, Chen SC, Li LP. Long-term results of palatal implantation for severe obstructive sleep apnea patients with prominent retropalatal collapse. *J Chin Med Assoc*. 2018;81(9):837-841. doi:10.1016/j.jcma.2018.01.012

19.	Huang TW, Cheng PW. Microdebrider-assisted extended uvulopalatoplasty: an effective and safe technique for selected patients with obstructive sleep apnea syndrome. *Arch Otolaryngol Head Neck Surg*. 2008;134(2):141-145. doi:10.1001/archoto.2007.27

20.	Hukins CA, Mitchell IC, Hillman DR. Radiofrequency tissue volume reduction of the soft palate in simple snoring. *Arch Otolaryngol Head Neck Surg*. 2000;126(5):602-606.

21.	Kwon JW, Kong TH, Ha TH, Park DJ. An absorbable thread suture technique to treat snoring. *Eur Arch Otorhinolaryngol*. 2016;273(5):1173-1178. doi:10.1007/s00405-015-3722-6

22.	Larrosa F, Hernandez L, Morello A, Ballester E, Quinto L, Montserrat JM. Laser-assisted uvulopalatoplasty for snoring: does it meet the expectations? *Eur Respir J*. 2004;24(1):66-70.

23.	Macdonald A, Drinnan M, Johnston A, et al. Evaluation of potential predictors of outcome of laser-assisted uvulopalatoplasty for snoring. *Otolaryngol Head Neck Surg*. 2006;134(2):197-203. doi:10.1016/j.otohns.2005.10.016

24.	Mantovani M, Carioli D, Torretta S, Rinaldi V, Ibba T, Pignataro L. Barbed snore surgery for concentric collapse at the velum: The Alianza technique. *J Craniomaxillofac Surg*. 2017;45(11):1794-1800. doi:10.1016/j.jcms.2017.08.007

25.	Maurer JT, Hein G, Verse T, Hormann K, Stuck BA. Long-term results of palatal implants for primary snoring. *Otolaryngol Head Neck Surg*. 2005;133(4):573-578. doi:10.1016/j.otohns.2005.07.027

26.	Maurer JT, Sommer JU, Hein G, Hormann K, Heiser C, Stuck BA. Palatal implants in the treatment of obstructive sleep apnea: a randomised, placebo-controlled single-centre trial. *Eur Arch Otorhinolaryngol*. 2012;269(7):1851-1856. doi:10.1007/s00405-011-1920-4

27.	Moffa A, Giorgi L, Nardelli D, et al. The potential impact of new remodelling intrapharynegal OSA surgery on sleep architecture: a preliminary investigation. *Sleep Breath*. 2025;29(1):38. doi:10.1007/s11325-024-03222-x

28.	Mora R, Salzano FA, Mora F, Guastini L. Outcomes of uvulopalatopharyngoplasty with harmonic scalpel after failure of continuous positive airway pressure in sleep apnea syndrome. *Acta Otolaryngol Stockh*. 2012;132(3):299-304. doi:10.3109/00016489.2011.637178

29.	Murphey AW, Nguyen SA, Fuller C, Weber AC, Camilon MP, Gillespie MB. T ran Q uill sling snoreplasty for snoring: A single‐arm pilot study for safety and effectiveness. *The Laryngoscope*. 2016;126(1):243-248. doi:10.1002/lary.25361

30.	Neruntarat C. Uvulopalatal flap for snoring on an outpatient basis. *Otolaryngol Head Neck Surg*. 2003;129(4):353-359.

31.	Neruntarat C. Long-term results of palatal implants for obstructive sleep apnea. *Eur Arch Otorhinolaryngol*. 2011;268(7):1077-1080. doi:10.1007/s00405-011-1511-4

32.	Nordgard S, Hein G, Stene BK, Skjostad KW, Maurer JT. One-year results: palatal implants for the treatment of obstructive sleep apnea. *Otolaryngol Head Neck Surg*. 2007;136(5):818-822.

33.	Rashwan MS, Khalafallah MA, Abdelsadek HM, Hassan MA, Emara TA. Is it important to stabilize palatopharyngeus muscle to pterygomandibular raphe under vision during surgery for OSA? *Eur Arch Otorhinolaryngol*. 2024;281(12):6687-6692. doi:10.1007/s00405-024-08924-2

34.	Server EA, Alkan Z, Yigit O, Yasak AG. Long-term results of pillar implant procedure. *Kulak Burun Bogaz Ihtis Derg*. 2016;26(5):258-264. doi:10.5606/kbbihtisas.2016.28924

35.	Sezen OS, Kaytanci H, Salepci B, et al. Palatal Pillar implants for the treatment of simple snoring. *Kulak Burun Bogaz Ihtis Derg*. 2009;19(5):239-245.

36.	Sher AE, Flexon PB, Hillman D, et al. Temperature-controlled radiofrequency tissue volume reduction in the human soft palate. *Otolaryngol--Head Neck Surg Off J Am Acad Otolaryngol-Head Neck Surg*. 2001;125(4):312-318.

37.	Steward DL, Huntley TC, Woodson BT, Surdulescu V. Palate implants for obstructive sleep apnea: Multi‐institution, randomized, placebo‐controlled study. *Otolaryngol Neck Surg*. 2008;139(4):506-510. doi:10.1016/j.otohns.2008.07.021

38.	Tatla T, Sandhu G, Croft CB, Kotecha B. Celon radiofrequency thermo-ablative palatoplasty for snoring - a pilot study. *J Laryngol Otol*. 2003;117(10):801-806.

39.	Terris DJ, Coker JF, Thomas AJ, Chavoya M. Preliminary findings from a prospective, randomized trial of two palatal operations for sleep-disordered breathing. *Otolaryngol Head Neck Surg*. 2002;127(4):315-323.

40.	Tschopp K, Mullis A, Knaus C, Tschopp S. A novel palato-pharyngeal implant system for the treatment of snoring and obstructive sleep apnea. *Eur Arch Otorhinolaryngol*. 2025;282(9):4877-4885. doi:10.1007/s00405-025-09545-z

41.	Tvinnereim M, Mitic S, Hansen RK. Plasma radiofrequency preceded by pressure recording enhances success for treating sleep-related breathing disorders. *Laryngoscope*. 2007;117(4):731-736.

42.	Ugur KS, Ark N, Kurtaran H, Kizilbulut G, Yuksel A, Gunduz M. Anterior palatoplasty for selected mild and moderate obstructive sleep apnea: preliminary results. *Eur Arch Otorhinolaryngol*. 2014;271(6):1777-1783. doi:10.1007/s00405-013-2701-z

43.	Walker RP, Levine HL, Hopp ML, Greene D, Pang K. Palatal implants: a new approach for the treatment of obstructive sleep apnea. *Otolaryngol Head Neck Surg*. 2006;135(4):549-554.

44.	Wassmuth Z, Mair E, Loube D, Leonard D. Cautery-assisted palatal stiffening operation for the treatment of obstructive sleep apnea syndrome. *Otolaryngol Head Neck Surg*. 2000;123(1 Pt 1):55-60.

45.	Al Bassiouny AMME. Posterior pillar flap palatoplasty: A new surgical technique for treatment of snoring: Initial experience. *J Med Sci*. 2009;9(3):118-125. doi:10.3923/jms.2009.118.125

46.	Daneshi A, Jahandideh H, Hosseinzadeh F. Titanium snoreplasty- A new surgical technique. *Iran J Otorhinolaryngol*. 2020;32(1):29-34. doi:10.22038/ijorl.2019.31930.2051

47.	Elbassiouny AM. Soft palatal webbing flap palatopharyngoplasty for both soft palatal and oropharyngeal lateral wall collapse in the treatment of snoring and obstructive sleep apnea: a new innovative technique without tonsillectomy. *Sleep Breath*. 2015;19(2):481-487. doi:10.1007/s11325-014-1067-9

48.	Elbassiuony AMME. Coblation-assisted soft palatal webbing flap uvulopalatoplasty: A new technique for the treatment of snoring. *Oper Tech Otolaryngol - Head Neck Surg*. 2012;23(3):211-218. doi:10.1016/j.otot.2012.04.001

49.	Goktas O, Solmaz M, Goktas G, Olze H. Long-term results in obstructive sleep apnea syndrome (OSAS) after laser-assisted uvulopalatoplasty (LAUP). *PLoS ONE*. 2014;9(6):e100211. doi:10.1371/journal.pone.0100211

50.	Lee LA, Yu JF, Lo YL, et al. Comparative Effects of Snoring Sound between Two Minimally Invasive Surgeries in the Treatment of Snoring: A Randomized Controlled Trial. Arias-Carrion O, ed. *PLoS ONE*. 2014;9(5):e97186. doi:10.1371/journal.pone.0097186

51.	Li KK, Powell NB, Riley RW, Troell RJ, Guilleminault C. Radiofrequency volumetric reduction of the palate: An extended follow-up study. *Otolaryngol Head Neck Surg*. 2000;122(3):410-414.

52.	Nordgard S, Stene BK, Skjostad KW. Soft palate implants for the treatment of mild to moderate obstructive sleep apnea. *Otolaryngol Head Neck Surg*. 2006;134(4):565-570.

53.	Picavet VA, Dellian M, Gehrking E, Sauter A, Hasselbacher K. Treatment of snoring using a non-invasive Er:YAG laser with SMOOTH mode (NightLase): a randomized controlled trial. *Eur Arch Oto-Rhino-Laryn*. 2023;280(1):307-312. doi:10.1007/s00405-022-07539-9

54.	Rotenberg BW, Luu K. Four-year outcomes of palatal implants for primary snoring treatment: a prospective longitudinal study. *Laryngoscope*. 2012;122(3):696-699. doi:10.1002/lary.22510

55.	Stuck BA. Radiofrequency-assisted uvulopalatoplasty for snoring: Long-term follow-up. *Laryngoscope*. 2009;119(8):1617-1620. doi:10.1002/lary.20488
